# Affordable Small Molecules as Promising Fluorescent Labels for Biomolecules

**DOI:** 10.3390/molecules29225237

**Published:** 2024-11-05

**Authors:** Raquel Eustáquio, João P. Prates Ramalho, Sílvia Arantes, António Candeias, Ana Teresa Caldeira, António Pereira

**Affiliations:** 1 HERCULES Laboratory, IN2PAST—Associate Laboratory for Research and Innovation in Heritage, Arts, Sustainability and Territory, University of Évora, Largo Marquês de Marialva 8, 7000-809 Évora, Portugal; raqueleustaquio1998@gmail.com (R.E.); jpcar@uevora.pt (J.P.P.R.); saa@uevora.pt (S.A.); candeias@uevora.pt (A.C.); atc@uevora.pt (A.T.C.); 2 Department of Chemistry and Biochemistry, School of Sciences and Technology, University of Évora, Rua Romão Ramalho 59, 7000-671 Évora, Portugal; 3 City U Macau Chair in Sustainable Heritage, Sino-Portugal Joint Laboratory of Cultural Heritage Conservation Science, University of Évora, Largo Marquês de Marialva 8, 7000-809 Évora, Portugal

**Keywords:** fluorescent labels, 4-diethylaminobenzaldehyde derivatives, biomolecules, RNA-FISH probes

## Abstract

Fluorescent labels, commonly used in highly sensitive analytical techniques for detecting and tracking biomolecules in critical fields like cellular biology, medicine, medicinal chemistry, and environmental science, are currently too expensive for routine use in standard applications, with most exhibiting small Stokes shifts. This limitation underscores the potential of 4-diethylaminobenzaldehyde derivatives as a cost-effective alternative for developing new, bright fluorophores with larger Stokes shifts. In this work, using 4-diethylaminobenzaldehyde as starting material, we developed a simple, cost-effective, and efficient synthetic strategy to produce new affordable small molecules as effective fluorescent labels for biomolecules. Density functional theory and time-dependent density functional theory calculations were also conducted to gain insights into the observed photophysical properties.

## 1. Introduction

The complex interconnections and coordinated regulations among bioactive molecules are fundamental in governing both physiological functions and the progression of certain diseases, including cancer, neurodegenerative disorders, and cardiovascular conditions [1]. In this context, chemical biology is a swiftly advancing domain that employs chemical techniques to unravel and control natural biological processes with remarkable precision [2,3,4,5]. Through interdisciplinary collaborations at the intersection of chemistry and biology, groundbreaking technologies have been developed to investigate biological systems, create diagnostic tools to enhance human well-being, and harness synthetic biology for medical and environmental advancements. These breakthroughs frequently depend on fluorescence-based imaging methodologies, that allow us to identify, observe, and analyze the morphological and dynamic aspects of biology at the molecular level through multi-dimensional approaches [4,5]. Due to their rapid response and high sensitivity, fluorescent probes offer several advantages for visualizing spatiotemporal alterations in biological systems and they have therefore become indispensable tools in the field of molecular biology and medicine, demonstrating their diagnostic and even clinical utility [6,7,8,9,10,11,12]. Fluorescent small organic molecules serve a multitude of purposes across a diverse range of experiments, including cellular staining, identifying specific bio-analytes, and monitoring biomolecules of significance. This makes them particularly appealing to organic chemists, given their numerous advantages over other imaging systems, such as their small size, chemical tunability, ease of use, and how they are often obtained through simple synthetic strategies [2,13]. The compact dimensions of fluorescent probes provide numerous benefits, such as minimal interference with the native functions of the target and extensive flexibility in both molecular design and application. Small fluorescent labels also offer significant practical benefits, enabling the optimization of fluorescence signals through the attachment of multiple fluorophores to a single biomolecule [14,15]. Typically, a donor-π bridge-acceptor structure (D-π-A) serves as the fundamental structural framework responsible for imparting fluorescence to small organic molecules [16,17]. Advancements over recent decades have demonstrated that the arrangement and characteristics of substituents in a D-π-A structure, promote the delocalization of the conjugated π electron system, producing derivatives with extraordinary photophysical and spectroscopic properties. Enhanced push–pull electronic effects in this kind of molecules, whose excitation involves intramolecular charge transfer (ICT), lead to a bathochromic (red) shift in both their UV-vis absorption and emission spectra [18,19,20]. Amine-reactive fluorescent labels are extensively utilized in various biological applications owing to their affinity for amino groups and their facile incorporation into biomolecules [21]. These fluorescent labels are indispensable in various modern scientific applications, including fluorescence microscopy, histochemistry, flow cytometry, cell tracing, receptor binding studies, direct and indirect immunochemistry, and fluorescence in situ hybridization (FISH) [22,23,24]. At present, the fluorescent labels are prohibitively expensive for regular use in routine applications and most of them have small Stokes shifts, less than 30 nm, such as fluorescein, rhodamine, oxazine, and cyanine. This limitation highlights the potential for 4-diethylaminobenzaldehyde derivatives to offer a cost-effective alternative for the development of new bright fluorophores with large Stokes shifts [25,26]. The design of molecules with donor-π bridge-acceptor (D-π-A) structures, as in this case, requires a strategic approach aimed at optimizing their electronic properties to increase fluorescence.

In this work, we intend to develop a simple and effective synthetic strategy to produce new cost-effective small molecules as fluorescent labels for biomolecules, using 4-diethylaminobenzaldehyde as a starting material. We also intend to evaluate the synthesized fluorescent labels as potentially effective fluorescent markers for biomolecules and compare their efficiency to Cy3^®^, one of the most used commercial fluorescent labels in a wide range of scientific applications. In the context of the FISH technique, six new fluorescent oligonucleotide probes were tested on microorganisms from the culture collection of the HERCULES Laboratory at the University of Évora. Three of these probes target the rRNA region of eukaryotic cells (EUK516), while the other three target the rRNA region of prokaryotic cells (EUB338). To provide additional insights to this study, Density Functional Theory (DFT) and Time-Dependent Density Functional Theory (TD-DFT) were employed to analyze the electronic, structural, and optical properties of the 4-styryl-*N*,*N*-diethylaniline derivatives in both the ground state and the lowest-lying singlet-excited states.

In the final analysis, the development of new fluorescent small organic molecules largely relies on trial and error or serendipitous discovery.

## 2. Results and Discussion

### 2.1. Synthesis and Characterization

**Scheme 1:**
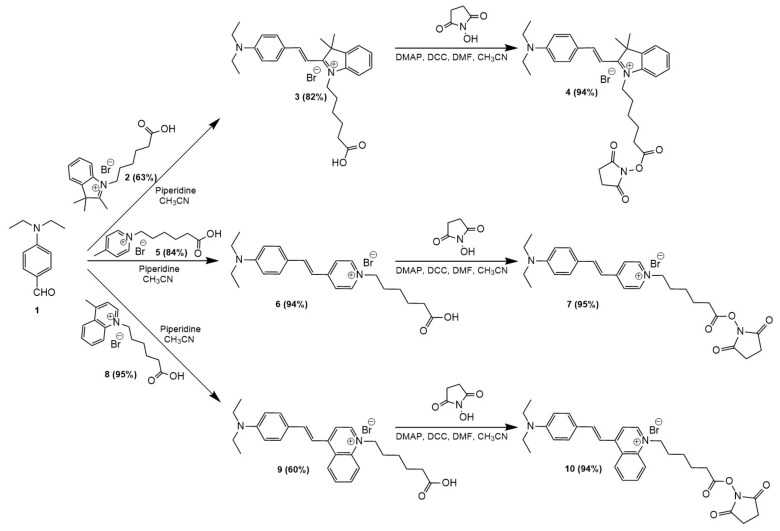
Synthetic route to 4-styryl-*N*,*N*-diethylaniline derivatives.

In this work, we develop a simple and cost-effective synthetic strategy, through three steps, to produce new cost-effective small molecules as promising fluorescent labels for biomolecules, using 4-diethylaminobenzaldehyde (**1**) as a starting material. The main synthetic approach for producing 4-styryl-*N*,*N*-diethylaniline derivatives relied on the high acidity of methyl protons present in indole, pyridine, and quinoline derivatives (compounds **2**, **5**, and **8**, respectively), which facilitate aldol condensation reactions. Scheme 1 illustrates the synthetic routes used to prepare the fluorescent labels for biomolecules **4**, **7**, and **10**, achieving very good to high yields.

The lower yield observed in the intermediate derivative **9** could be attributed to a decrease in acidity of the methyl protons within the quinoline derivative **8**. This decrease may have resulted from enhanced electronic delocalization across the molecular structure, facilitated by the additional presence of the phenyl group, when compared with pyridine derivative **5**. The highly stereoselective and efficient aldol condensation reaction between the 4-diethylaminobenzaldehyde **1** and the indole, pyridine, and quinoline derivatives, with effective electron-accepting groups, allowed the synthesis of intermediates **3**, **6,** and **9**. The presence, in these intermediates, of an electron-donating group (EDG) and an electron-accepting group (EWG), would clearly increase both the π-delocalization and the push–pull nature of the chromophore. The abovementioned new products **4**, **7,** and **10** (Scheme 1) were readily isolated via silica column chromatography and fully characterized using NMR spectroscopy (1D and 2D), as well as HRMS (SI). All spectral data align well with the proposed structures.

### 2.2. Photophysical Characterization

The photophysical properties of the synthesized 4-styryl-*N*,*N*-diethylaniline derivatives (compounds **4**, **7,** and **10**) were analyzed, and their absorption and emission characteristics, along with fluorescence quantum yields, are summarized in Table 1.

The extension of the π-conjugated system in these molecules is essential in facilitating the effective intramolecular charge transfer (ICT) process of the emissive excited state, improving their photophysical properties, such as high molar extinction coefficients and, in the case of compounds **7** and **10**, large Stokes shifts. Derivative **4** exhibits a small Stokes shift, likely due to efficient energy relaxation, limited structural flexibility, or similar energy levels in their electronic transitions. The possible elimination of the spectral overlap between absorption and emission, in fluorophores with large Stokes shifts, allows us to eliminate the quenching process and reduce interference, providing the effective detection of the fluorescence emission. As expected, the 4-styryl-*N*,*N*-diethylaniline derivatives **4**, **7,** and **10** exhibit absorption maxima at significantly longer wavelengths compared to 4-diethylaminobenzaldehyde (**1**). This is attributed to the intramolecular charge transfer (ICT) effect resulting from the conjugation of the electron-donating NEt_2_ group and the styryl group at position 4, which is also conjugated with the electron-withdrawing groups. The most intense absorption band, occurring in the range of 390–690 nm, is likely due to the delocalized π→π* transition of the aromatic moieties present in the 4-styryl-*N*,*N*-diethylaniline derivatives. The shorter or less extensive conjugated system in derivative **7**, compared to the systems in derivatives **4** and **10**, can create a higher energy gap between the ground and excited states, resulting in absorption at shorter wavelengths. The molar coefficients and the fluorescence quantum yields were strongly affected, both in the same direction, by the nature of the EWGs presents in the 4-styryl-*N*,*N*-diethylaniline derivatives. The analysis of Table 1 confirms that the indole moiety enhances both the extinction coefficient and fluorescence quantum yield compared to the lower values observed with the quinoline moiety. This significant decrease in fluorescence quantum yield is likely attributed to non-radiative relaxation or competing deactivation pathways occurring in the quinoline moiety. This difference may also stem from the varying resonance energy effects of the electron-withdrawing groups (EWGs).

### 2.3. Theoretical Calculations

**Figure 1:**
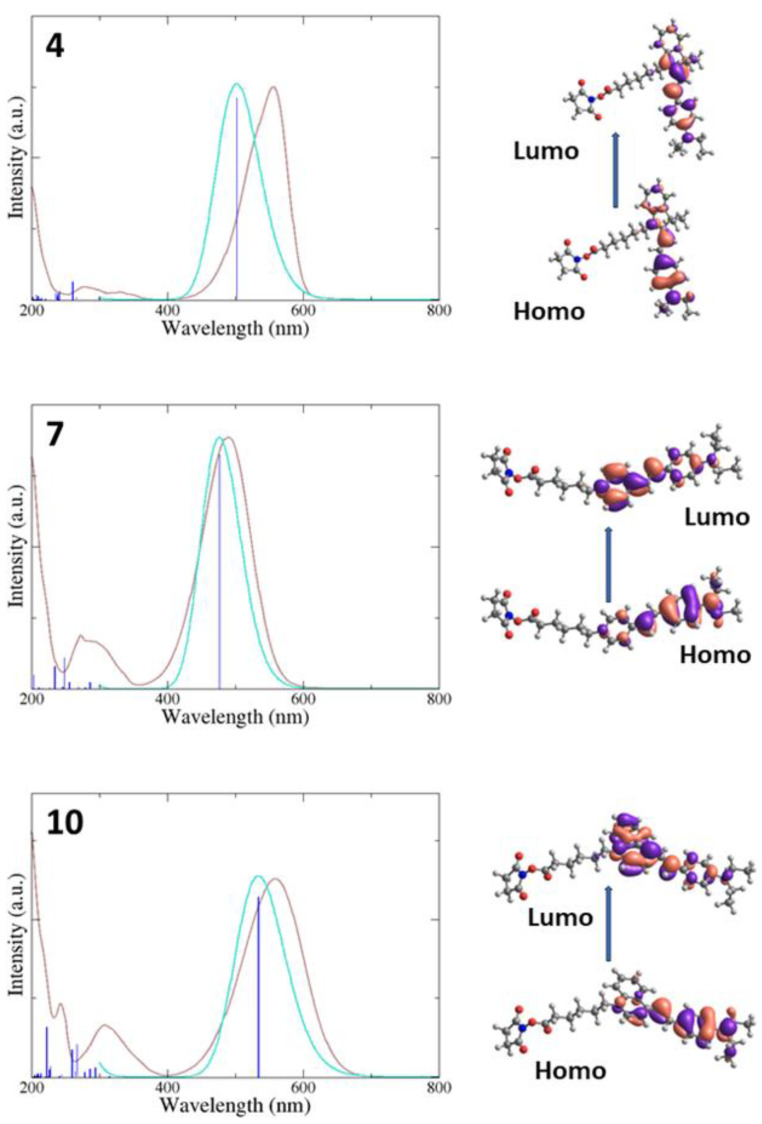
Experimental spectra (violet) compared with the calculated spectra (light blue) and electronic transition energies (dark blue). Contour plots of the molecular orbitals involved in the lowest energy excitation on the right.

The lower energy band of the spectra comprises a single, well-separated S1 excitation. This band is mainly of HOMO → LUMO nature and is strongly dipole-allowed, with an oscillator strength (ff) greater than 1, for all the studied compounds (Figure 1 and Table S1). For all the compounds, both the HOMO and LUMO orbitals display minimal involvement around the attached reactive group, suggesting that this group does not participate in the π-conjugation system and remains uninvolved in the excitation process. This is a valuable aspect for maintaining the dye’s fluorescence properties when attached to a biomolecule. Focusing on the HOMO states, the prominent characteristic observed is that these orbitals exhibit greater localization in the 4-styryl-*N*,*N*-diethylaniline moiety for the three compounds while the LUMO orbitals spread over the indole, pyridine, and quinoline groups. This is more pronounced for compound **10** and less noticeable for compound **4**.

A thorough definition of the excitation type can be achieved by analyzing the orbitals involved or the electronic density difference that occurs upon excitation. The S_0_ → S_1_ transition can be attributed to π→π* electron transitions and is primarily of HOMO→LUMO character for all the compounds. The strongest absorption band, in the region of 390–690 nm, is produced from the delocalized π—π* transition of aromatic moieties present in the 4-styryl-*N*,*N*-diethylaniline derivatives. The less extensive conjugated system in derivative **7**, compared to derivatives **4** and **10**, results in a higher energy gap between the ground and the excited states, leading to absorption at shorter wavelengths. An illustrative depiction of the charge rearrangement occurring during the S_0_→S_1_ transitions is provided in Figure S1. The figure shows the difference in electronic densities between the states involved in the transition, clearly indicating the regions of the chromophore that lose or gain electronic charge upon excitation. It is very clear that for compounds **7** and **10** there are large rearrangements of charge density with a clear decrease and increase in density in distinct regions of the molecule, thereby allowing the transition to be classified as intramolecular charge transfer (ICT). To quantify the charge transfer distance, the Δr index [27] was used to analyze the nature of these electronic transitions.

Transitions with a high Δr index reflect a strong ICT character, typically classified as such when exceeding the widely accepted threshold of 2.0 Å. Following this criterion, all the S_1_ excitations can be classified as ICT. However, the index value is much larger, indicating a much larger distance displacement of the charge upon excitation for compounds **7** and **10**, while for compound **4**, it is at the ICT classification limit. Another parameter employed to characterize the nature of electronic transitions is the lambda index Λ, which is a measure of the overlap between the hole and electron states during electronic excitations. Notably, the Λ indices are almost inversely proportional to the Δr indices, since a greater hole–electron overlap generally indicates a shorter separation distance between them. Following the trend of the Δr observed value, the Λ values are significantly smaller for compounds **7** and **10** compared to compound **4**. The molecular configurations of the 4-styryl-*N*,*N*-diethylaniline derivatives in their ground state are depicted in Figure S1. In their ground state, deviations from planarity (~19.4° and 8.1°) are observed in derivatives **4** and **10**, respectively, namely between the planes of the two rings, whereas compound **7** appear predominantly planar (Table S2). Upon entering the S_1_ electronic excited state, the overall structural arrangement of compound **7**, already planar, remains relatively stable, with compound **4** undergoing a rotation of 2.9° toward planarity and compound **10** becoming near planar. The transition from the S_0_ to the S_1_ state is marked by a significant decrease in bond length alternation (BLA) along the p bridge, signifying the average length difference between a single and adjacent double bond, which diminishes notably across all compounds. In the S_0_ state, BLA values range from 0.04 Å for compound **4** to 0.07 Å for compound **7**. In contrast, in the excited state S_1_, the BLA undergoes a substantial reduction, reaching 0.00 Å for compound **4** and peaking at 0.02 Å for compound **10**. The emission characteristics of the dyes, calculated from the optimized geometry of the S_1_ state, are presented in Table S1. The emission wavelengths for the S_1_→S_0_ transition are consistently shorter than those of the S_0_→S_1_ absorption process, which is based on the ground-state optimized geometry. In the electronic excited state S_1_, geometric changes enhance π-conjugation delocalization, leading to a narrowed HOMO–LUMO energy gap. As a result, after vertical excitation, the excited state undergoes structural relaxation before emission, producing a lower emission energy than the excitation energy [28,29]. Kasha’s rule states that fluorescence generally originates from the lowest excited state, with higher electronic excited states usually not playing a direct role in the emission of excited fluorophores [30]. Therefore, the emissions from the dyes examined in this study are expected to originate from the S_1_ state. The smaller Stokes shift noted for derivative **4** can be explained by the slight variations in the geometries between the excited state S_1_ and the ground state S_0_. As a result, the HOMO–LUMO energy gap experiences a smaller decrease compared to the other derivatives. Notably, compound 4 exhibits a rotation of 2.9°, maintaining a distinctly non-planar structure in the S_1_ excited state, while compound **10** approaches a nearly planar configuration. In contrast, compound **7** retains an almost planar conformation but undergoes a considerable decrease in BLA values, resulting in a smaller energy gap. Based on the geometry optimization of the lowest excited state S_1_, the theoretical fluorescence lifetime of the excited states can be determined using Einstein’s equation for the transition probabilities of spontaneous emissions.

$$\tau =\frac{c^{3}}{2E^{2}f}$$

Here, *τ* represents the fluorescence lifetime, *c* is the speed of light in a vacuum, *E* denotes the transition energy, and *f* corresponds to the oscillator strength for the S_1_→S_0_ transition. The calculated fluorescence lifetimes for the dyes are presented in Table S3. For all dyes examined, the values are significantly shorter than 10 ns, which is typical for emissive states of organic fluorophores, whereas longer radiative lifetimes are associated with nonradiative states.

### 2.4. RNA-FISH Performance of the Synthesized Oligonucleotide Probes

The two following microorganisms were chosen as biological models: (i) the yeast *Saccharomyces cerevisiae* and (ii) the bacterium *Bacillus* sp.

#### 2.4.1. Hybridization of *Saccharomyces cerevisiae*

**Figure 2:**
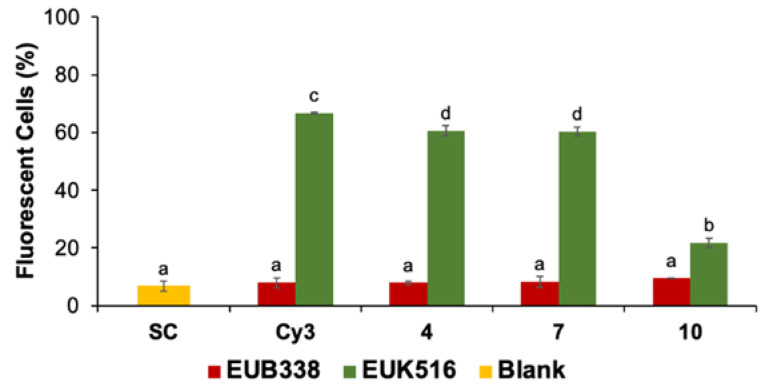
Percentage of fluorescent cells from *Saccharomyces cerevisiae* cells labeled with EUB338 and EUK516 probes with different markers (**4**, **7**, **10,** and Cy3) and their respective blank. In each assay, 1000 cells were analyzed in triplicate. Values represented correspond to the average of the flow cytometry measurements and error bars to standard deviation (±SD). In each plot, different letters located over the error bars indicate significant differences (*p* < 0.05).

The results of the flow cytometry analysis of the hybridization of *Saccharomyces cerevisiae* (SC) cells with both commercial and synthesized oligonucleotide probes are presented in Figure S2. After a comprehensive analysis of these results, yeast cells labeled with EUK516 oligonucleotide probes demonstrate significantly higher fluorescence levels than the autofluorescence observed. This finding aligns with expectations, as these probes are designed to specifically target the rRNA region of eukaryotic cells (Figure S2A,C,E,G). Cells hybridized with the EUK516-Cy3 probe exhibit significantly higher fluorescence intensity compared to the autofluorescence of unlabeled *Saccharomyces cerevisiae* (SC) cells (Figure S2A). This fluorescence signal indicates the successful hybridization of the EUK516-Cy3 probe, demonstrating its effective binding capacity. In contrast, cells treated with the EUB338-Cy3 probe emitted fluorescence signals comparable to the autofluorescence observed in unlabeled cells as anticipated, indicating a lack of hybridization (Figure S2B). Therefore, this probe adequately performs its function as a negative control in experiments involving eukaryotic cells, validating that, under the experimental conditions, cellular autofluorescence enables specific and reliable detection. In the case of cells hybridized with the EUK516 probes with the synthesized fluorescent labels, only the cells labelled with the EUK516-(**4**) and EUK516-(**7**) probes show fluorescence signals (Figure S2C,E), indicating that these probes have a higher hybridization rate than the EUK516-(**10**) probe (Figure S2G). This probe shows unpromising results, as it shows similar fluorescence intensities to the negative control (EUB338-(**10**)) and to yeast autofluorescence (SC). In contrast, the cells hybridized with the EUB338-(**4**), EUB338-(**7**), and EUB338-(**10**) probes exhibited fluorescence signals identical to the autofluorescence of the yeast cells, as expected (Figure S2D,F,H). The percentage of fluorescent cells from *Saccharomyces cerevisiae* (SC) hybridized with the synthesized oligonucleotide probes EUK516-(**4**) and EUK516-(**7**) show a high value, close to the values of the commercial probe EUK516-Cy3. These values indicate that these probes have a high specificity for labeling SC cells. On the other hand, in the cells hybridized with the EUK516-(**10**) probe, a low percentage of fluorescent cells of less than 25% was observed, which indicates that this probe has little specificity for labeling these cells. Analysis of the hybridization of *Saccharomyces cerevisiae* cells with the oligonucleotide probes EUB338-(**4**), EUB338-(**7**), and EUB338-(**10**) revealed that they showed identical values for the percentage of fluorescent cells to those of unmarked *Saccharomyces cerevisiae* cells and cells labeled with the EUB338-Cy3 probe. This result once again reinforces the lack of specificity of these probes for eukaryotic cells (Figure 2). Epifluorescence microscopy confirms the results obtained by flow cytometry, highlighting that the oligonucleotide probes EUK516-Cy3, EUK516-(**4**), and EUK516-(**7**) shown in Figure S3A,C,E, show more intense signals. Regarding the results of the hybridization tests of *Saccharomyces cerevisiae* cells with the EUB338 oligonucleotide probes, no fluorescence signal was detected (Figure S3B,D,F).

#### 2.4.2. Hybridization of *Bacillus* sp.

**Figure 3:**
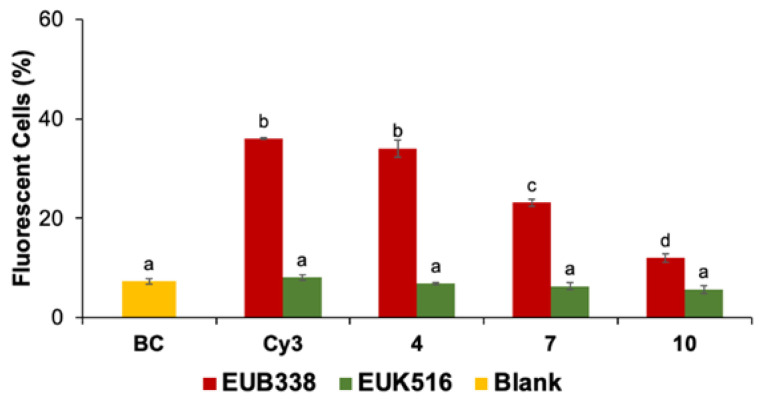
The percentage of fluorescent *Bacillus* sp. cells labeled with EUB338 and EUK516 probes, each with different markers (**4**, **7**, **10**, and Cy3), along with their respective controls, is shown. In each assay, 1000 cells were analyzed in triplicate. The values presented represent the average flow cytometry measurements, with error bars indicating the standard deviation (±SD). In each plot, distinct letters above the error bars denote significant differences (*p* < 0.05).

The flow cytometry results demonstrating the hybridization of *Bacillus* sp. (BA) cells with both commercial and synthesized oligonucleotide probes are shown in Figure S4. Bacterial cells exhibit more intense fluorescence than autofluorescence when labeled with the EUB338 oligonucleotide probes, since these probes target the rRNA region of prokaryotic cells (Figure S4A,C,E,G). Cells hybridized with the EUB338-Cy3 probe emitted a higher fluorescence intensity than the autofluorescence of unlabeled bacterial cells (BA) (Figure S4A). This fluorescence indicates that the EUB338-Cy3 probe has high hybridization rates. In contrast, cells hybridized with the EUK516-Cy3 probe exhibited fluorescence intensities (FI) like the autofluorescence of unlabeled cells, indicating no hybridization (Figure S4B). In the case of cells hybridized with the EUB338 probes with the synthesized labels, it should be noted that the cells labeled with the EUB338-(**4**) and EUB338-(**7**) probes (Figure S4C,E) show greater fluorescence than the cells hybridized with the EUB338-(**10**) probe (Figure S4G). The EUB338-(**10**) probe shows less promising results, since the cells labeled with this probe show the same fluorescence intensity as the autofluorescence of unlabeled yeast cells (Figure S4H). The percentage of fluorescent cells from *Bacillus* sp. (BA) hybridized with the synthesized oligonucleotide probes EUB338-(**4**) and EUB338-(**7**) results in a notable increase in the percentage of fluorescent cells compared to unlabeled cells (Figure 3). When *Bacillus* sp. cells were hybridized with the synthesized oligonucleotide probe EUB338-(4), there was a statistically significant increase in the percentage of fluorescent cells (*p* < 0.05) compared to the control cells hybridized with the synthesized oligonucleotide probes EUB338-(**7**) and EUB338-(**10**). The EUB338-(**4**) probe shows statistically identical values for the percentage of fluorescent cells to the cells hybridized with the commercial probe EUB338-Cy3. On the other hand, cells labeled with the EUB338-(**10**) probe showed a low percentage of fluorescent cells, less than 20%. This result indicates that the EUB338-(**10**) probe has low specificity for labeling prokaryotic cells. Once again, the epifluorescence microscopy observations validate the results obtained by flow cytometry, since the oligonucleotide probes EUB338-Cy3, EUB338-(**4**), and EUB338-(**7**) (Figure S5A,C,E) show higher fluorescence intensities. Conversely, in the case of the hybridization tests of *Bacillus* cells with the probes EUK516-Cy3, EUK516-(**4**), EUK516-(**7**), and EUK516-(**10**), no fluorescence signal was detected, as anticipated (Figure S5B,D,F,H). These findings further confirm that these probes lack specificity for prokaryotic cells, emphasizing the selective binding of the synthesized fluorophores to their respective oligonucleotide probes, thus avoiding nonspecific interactions with the cells.

## 3. Experimental Section

### 3.1. Materials and Equipment

All starting materials and reagents were of analytical grade, obtained from Aldrich (Waltham, MA, USA), and used as received without further purification. Organic solvents were dried with appropriate desiccants and distilled prior to use. UV-vis absorption spectra were obtained using acetonitrile (CH_3_CN) as the solvent on a Thermo Electron Corporation (Waltham, MA, USA) spectrophotometer, model Nicolet Evolution 300 (Waltham, MA USA). Fluorescence spectra were measured with a PerkinElmer Model LS 55 spectrophotometer (Waltham, MA USA). All emission spectra were collected using a slit bandwidth of 5.0 nm for both excitation and emission, along with correction files. Spectroscopic measurements were conducted in 3 mL quartz fluorescence cuvettes with a 1 cm optical path length at a temperature of 21 °C. Nuclear magnetic resonance (NMR) spectra were recorded at 400 MHz for ^1^H NMR and 100 MHz for ^13^C NMR using a Bruker Advance III spectrometer (Billerica, MA, USA). Deuterated methanol (CD_3_OD) was employed as the solvent. Chemical shifts (δ) are reported in parts per million (ppm), coupling constants (J) are expressed in Hertz (Hz), and relative intensities are indicated by the number of protons (H). Multiplicities are denoted as singlet (s), doublet (d), double-doublet (dd), triplet (t), quadruplet (q), and multiplet (m). FTMS-ESI mass spectra were recorded using a Thermo Scientific Q Exactive Orbitrap Mass Spectrometer (Waltham, MA, USA).

### 3.2. Synthesis

#### 3.2.1. Synthesis of (1-(5-Carboxypentyl)-2,3,3-trimethyl-3*H*-indol-1-ium Bromide (**2**)

A mixture of 2,3,3-trimethyl-3*H*-indole (500 mg, 3.14 mmol, 1.0 eq) and 6-bromohexanoic acid (919 mg, 4.71 mmol, 1.5 eq) in dry DMF (10 mL) was stirred at 100 °C for a period of 24 h. The reaction was continuously monitored by TLC, using CHCl_3_/CH_3_OH (8:2) as the eluent. The reaction mixture was evaporated to dryness and the residue purified by flash chromatography, using CHCl_3_, CHCl_3_/CH_3_OH (9:1) and CHCl_3_/CH_3_OH (8:2) as eluents, to yield (1-(5-carboxypentyl)-2,3,3-trimethyl-3*H*-indol-1-ium bromide as a rose solid (700 mg, 63%). ^1^H NMR (400 MHz, CD_3_OD, δ, ppm): 1.50–1.57 (2H, m, H-13), 1.62 (6H, s, H-8, H-9), 1.66–1.74 (2H, m, H-14), 1.95–2.03 (2H, m, H-12), 2.33 (2H, t, *J* = 7.3, H-15), 2.71 (3H, s, H-10), 4.53 (2H, t, *J* = 7.3, H-11), 7.63–7.68 (2H, m, H-5, H-6), 7.77–7.79 (1H, m, H-7), 7.88–7.92 (1H, m, H-4). ^13^C NMR (100 MHz, CD_3_OD, δ, ppm): 22.8 (C-10), 25.4 (C-14), 27.1 (C-13), 28.6 (C-8, C-9), 34.4 (C-12), 35.4 (C-15), 49.2 (C-11), 55.9 (C-3), 116.6 (C-7), 124.7 (C-4), 130.5 (C-5), 131.2 (C-6), 142.5 (C-7a), 143.4 (C-3a), 177.1 (C-2), 197.9 (COOH).

#### 3.2.2. Synthesis of (*E*)-1-(5-Carboxypentyl)-2-(4-(diethylamino)styryl)-3,3-dimethyl-3*H*-indol-1-ium Bromide (**3**)

A mixture of compound **2** (50 mg, 0.141 mmol, 1.0 eq), 4-diethylaminobenzaldehyde (25 mg, 0.141 mmol, 1.0 eq), and piperidine (28 µL, 0.282 mmol, 2.0 eq) in dry acetonitrile (5 mL) was stirred at 85 °C for 1 h. The reaction was monitored continuously by TLC, using a CHCl_3_/CH_3_OH/H_2_O (65:20:2) eluent. After evaporation to dryness, the residue was purified by flash chromatography, using CHCl_3_/CH_3_OH/H_2_O (65:20:2) as eluent, to yield (*E*)-1-(5-carboxypentyl)-2-(4-(diethylamino)styryl)-3,3-dimethyl-3*H*-indol-1-ium bromide as a pink solid (59 mg, 82%). ^1^H NMR (400 MHz, CD_3_OD, δ, ppm): 1.27 (6H, t, *J* = 7.1, N(CH_2_*CH*_3_)_2_), 1.49–1.57 (2H, m, H-13), 1.67–1.74 (2H, m, H-14), 1.80 (6H, s, H-8, H-9), 1.89–1.97 (2H, m, H-12), 2.23 (2H, t, *J* = 7.3, H-15), 3.60 (4H q, *J* = 7.1, N(*CH*_2_CH_3_)_2_), 4.45 (2H, t, *J* = 7.3, H-11), 6.91 (2H, d, *J*_20,19_ = 9.2, H-20, H-22), 7.16 (1H, d, *J*_10,17_ = 15.5, H-10), 7.47 (1H, dt, *J*_5,4_ = 7.4, H-5), 7.54 (1H, dt, *J*_6,7_ = 7.9, H-6), 7.61 (1H, d, *J*_7,6_ = 7.9, H-7), 7.65 (1H, dd, *J*_4,5_ = 7.4, H-4), 7.93 (2H, d, *J*_19,20_ = 8.4, H-19, H-23), 8.29 (1H, d, *J*_17,16_ = 15.5, H-17). ^13^C NMR (100 MHz, CD_3_OD, δ, ppm): 12.9 (C-25, C-27), 26.6 (C-14), 27.4 (C-13), 27.5 (C-8, C-9), 28.9 (C-12), 37.3 (C-15), 46.1 (C-24, C-26), 46.3 (C-11), 52.4 (C-3), 104.6 (C-10), 113.6 (C-20, C-22), 114.3 (C-7), 123.6 (C-18), 123.8 (C-4), 128.8 (C-5), 130.2 (C-6), 135.1 (C-19, C-23), 142.7 (C-7a), 144.0 (C-3a), 155.0 (C-21), 156.3 (C-17), 180.7 (C-2, COOH). UV λ_max_ (nm, CH_3_CN): 280, 556. ε (cm^−1^·M^−1^): 90,000. Φ_F_ = 0.57.

#### 3.2.3. Synthesis of (*E*)-2-(4-(Diethylamino)styryl)-1-(6-((2,5-dioxopyrrolidin-1-yl)oxy)-6-oxohexyl)-3,3-dimethyl-3*H*-indol-1-ium Bromide (**4**)

A mixture of compound **3** (117 mg, 0.228 mmol, 1.0 eq), DCC (56 mg, 0.273 mmol, 1.2 eq), and DMAP (3 mg, 0.023 mmol, 0.1 eq) in dry DMF (2 mL) and dry acetonitrile (8 mL) was stirred at room temperature for 5 min. After this period, *N*-hydroxysuccinimide (31 mg, 0.273 mmol, 1.2 eq) was added, and the reaction was stirred at room temperature for an additional 6 h. The progress of the reaction was monitored by TLC, using a CHCl_3_/CH_3_OH/H_2_O (65:20:2) eluent. Following evaporation to dryness, the residue was purified by flash chromatography, using CHCl_3_, CHCl_3_/CH_3_OH (99:1), CHCl_3_/CH_3_OH (95:5), CHCl_3_/CH_3_OH (9:1) and CHCl_3_/CH_3_OH (8:2) as eluents, to yield (*E*)-2-(4-(diethylamino)styryl)-1-(6-((2,5-dioxopyrrolidin-1-yl)oxy)-6-oxohexyl)-3,3-dimethyl-3*H*-indol-1-ium bromide as a purple solid (131 mg, 94%). ^1^H NMR (400 MHz, CD_3_OD, δ, ppm): 1.27 (6H, t, *J* = 7.1, N(CH_2_*CH*_3_)_2_), 1.63–1.69 (2H, m, H-13), 1.80 (6H, s, H-8, H-9), 1.82–1.89 (2H, m, H-14), 1.93–2.00 (2H, m, H-12), 2.67 (2H, t, *J* = 7.3, H-15), 2.78 (4H, s, H-29, H-30), 3.61 (4H q, *J* = 7.1, N(*CH*_2_CH_3_)_2_), 4.47 (2H, t, *J* = 7.3, H-11), 6.90 (2H, d, *J*_20,19_ = 9.2, H-20, H-22), 7.16 (1H, d, *J*_10,17_ = 15.5, H-10), 7.47 (1H, dt, *J*_5,4_ = 7.4, H-5), 7.54 (1H, dt, *J*_6,7_ = 7.9, H-6), 7.61 (1H, d, *J*_7,6_ = 7.9, H-7), 7.65 (1H, dd, *J*_4,5_ = 7.4, H-4), 7.94 (2H, d, *J*_19,20_ = 8.4, H-19, H-23), 8.29 (1H, d, *J*_17,16_ = 15.5, H-17). ^13^C NMR (100 MHz, CD_3_OD, δ, ppm): 12.9 (C-25, C-27), 26.5 (C-29, C-30), 26.8 (C-14), 27.5 (C-13, C-8, C-9), 28.5 (C-12), 31.4 (C-15), 46.1 (C-24, C-26), 46.2 (C-11), 52.4 (C-3), 104.7 (C-10), 113.5 (C-20, C-22), 114.3 (C-7), 123.6 (C-18), 123.8 (C-4), 128.8 (C-5), 130.2 (C-6), 135.8 (C-19, C-23), 142.6 (C-7a), 143.9 (C-3a), 155.0 (C-21), 156.4 (C-17), 170.2 (C-16), 171.8 (C-28, C-31). FTMS(+) calc. for C_32_H_40_O_4_N_3_ [M+H]^+^ 530.3013 found 530.3012. UV λ_max_ (nm, CH_3_CN): 280, 556. ε (cm^−1^·M^−1^): 90,000. Φ_F_ = 0.57.

#### 3.2.4. Synthesis of 1-(5-Carboxypentyl)-4-methylpyridin-1-ium Bromide (**5**)

A mixture of 4-methylpyridine (500 mg, 5.37 mmol, 1.0 eq) and 6-bromohexanoic acid (1.6 g, 8.05 mmol, 1.5 eq) in dry DMF (10 mL) was stirred at 100 °C for a period of 2 h and 30min. The reaction was monitored continuously by TLC using CHCl_3_/CH_3_OH (8:2) as the eluent. After evaporation to dryness, the residue was purified by flash chromatography, with CHCl_3_, CHCl_3_/CH_3_OH (9:1) and CHCl_3_/CH_3_OH (8:2) as eluents, to yield 1-(5-carboxypentyl)-4-methylpyridin-1-ium bromide as a white solid (1.3 g, 84%). ^1^H NMR (400 MHz, CD_3_OD, δ, ppm): 1.38–1.46 (2H, m, H-10), 1.64–1.71 (2H, m, H-11), 1.99–2.07 (2H, m, H-9), 2.32 (2H, t, *J* = 7.2, H-12), 2.69 (3H, s, H-7), 4.61 (2H, t, *J* = 7.4, H-8), 7.95 (2H, d, *J*_3,2_ = 6.9, H-3, H-5), 8.85 (2H, d, *J*_2,3_ = 6.9, H-2, H-6). ^13^C NMR (100 MHz, CD_3_OD, δ, ppm): 22.0 (C-7), 25.2 (C-11), 26.5 (C-10), 32.0 (C-9), 34.4 (C-12), 61.9 (C-8), 129.9 (C-3, C-5), 144.9 (C-2, C-6), 161.2 (C-4), 177.1 (COOH).

#### 3.2.5. Synthesis of (*E*)-1-(5-Carboxypentyl)-4-(4-(diethylamino)styryl)pyridin-1-ium Bromide (**6**)

A mixture of compound **5** (100 mg, 0.347 mmol, 1.0 eq), 4-diethylaminobenzaldehyde (62 mg, 0.347 mmol, 1.0 eq), and piperidine (69 µL, 0.694 mmol, 2.0 eq) in dry acetonitrile (10 mL) was stirred at 85 °C for 4 h. The reaction was continuously monitored by TLC using CHCl_3_/CH_3_OH/H_2_O (65:20:2) as the eluent. After evaporation to dryness, the residue was purified by flash chromatography, using CHCl_3_/CH_3_OH (8:2) and CHCl_3_/CH_3_OH/H_2_O (65:35:5) as eluents, to yield (*E*)-1-(5-carboxypentyl)-4-(4-(diethylamino)styryl)pyridin-1-ium bromide as a red solid (146 mg, 94%). ^1^H NMR (400 MHz, CD_3_OD, δ, ppm): 1.20 (6H, t, *J* = 7.1, N(CH_2_*CH*_3_)_2_), 1.35–1.41 (2H, m, H-10), 1.62–1.70 (2H, m, H-11), 1.92–2.00 (2H, m, H-9), 2.24 (2H, t, *J* = 7.2, H-12), 3.47 (4H q, *J* = 7.1, N(*CH*_2_CH_3_)_2_), 4.39 (2H, t, *J* = 7.4, H-8), 6.74 (2H, d, *J*_17,16_ = 9.0, H-17, H-19), 7.03 (1H, d, *J*_7,14_ = 15.9, H-7), 7.58 (2H, d, *J*_16,17_ = 9.0, H-16, H-20), 7.80 (1H, d, *J*_14,7_ = 15.9, H-14), 7.94 (2H, d, *J*_3,2_ = 6.9, H-3, H-5), 8.55 (2H, d, *J*_2,3_ = 6.9, H-2, H-6). ^13^C NMR (100 MHz, CD_3_OD, δ, ppm): 12.9 (C-22, C-24), 26.0 (C-11), 26.8 (C-10), 31.9 (C-9), 36.8 (C-12), 45.5 (C-21, C-23), 60.9 (C-8), 112.6 (C-17, C-19), 117.1 (C-7), 123.4 (C-15), 123.6 (C-3, C-5), 132.0 (C-16, C-20), 144.4 (C-2, C-6), 144.4 (C-14), 151.5 (C-18), 156.3 (C-4), 180.2 (COOH). UV λ_max_ (nm, CH_3_CN): 273, 490. ε (cm^−1^·M^−1^): 64,000. Φ_F_ = 0.30.

#### 3.2.6. Synthesis of (*E*)-4-(4-(Diethylamino)styryl)-1-(6-((2,5-dioxopyrrolidin-1-yl)oxy)-6-oxohexyl)pyridin-1-ium Bromide (**7**)

A mixture of compound **6** (188 mg, 0.420 mmol, 1.0 eq), DCC (104 mg, 0.504 mmol, 1.2 eq), and DMAP (5 mg, 0.042 mmol, 0.1 eq) in dry DMF (2 mL) and dry acetonitrile (8 mL) was stirred at room temperature for 5 min. After this time, *N*-hydroxysuccinimide (58 mg, 0.504 mmol, 1.2 eq) was added, and the reaction was stirred at room temperature for an additional 6 h. The progress of the reaction was monitored by TLC using CHCl_3_/CH_3_OH/H_2_O (65:20:2) as the eluent. Following evaporation to dryness, the residue was purified by flash chromatography, using CHCl_3_, CHCl_3_/CH_3_OH (99:1), CHCl_3_/CH_3_OH (95:5), CHCl_3_/CH_3_OH (90:10), and CHCl_3_/CH_3_OH (80:20) as eluents, yielding (*E*)-4-(4-(diethylamino)styryl)-1-(6-((2,5-dioxopyrrolidin-1-yl)oxy)-6-oxohexyl)pyridin-1-ium bromide as a red solid. (217 mg, 95%). ^1^H NMR (400 MHz, CD_3_OD, δ, ppm): 1.20 (6H, t, *J* = 7.1, N(CH_2_*CH*_3_)_2_), 1.45–1.54 (1H, m, H-10), 1.77–1.85 (1H, m, H-11), 1.94–2.04 (2H, m, H-9), 2.68 (2H, t, *J* = 7.0, H-12), 2.82 (3H, s, H-26, H-27), 3.47 (4H q, *J* = 7.1, N(*CH*_2_CH_3_)_2_), 4.43 (2H, t, *J* = 7.3, H-8), 6.76 (2H, d, *J*_17,16_ = 8.8, H-17, H-19), 7.06 (1H, d, *J*_7,14_ = 16, H-7), 7.59 (2H, d, *J*_16,17_ = 8.9, H-16, H-20), 7.83 (1H, d, *J*_14,7_ = 16, H-14), 7.96 (2H, d, *J*_3,2_ = 7.2, H-3, H-5), 8.56 (1H, d, *J*_2,3_ = 7.2, H-2, H-6). ^13^C NMR (100 MHz, CD_3_OD, δ, ppm): 12.9 (C-22, C-24), 25.2 (C-26, C-27), 26.0 (C-11), 26.9 (C-10), 31.9 (C-9), 34.3 (C-12), 45.5 (C-21, C-23), 60.8 (C-8), 112.7 (C-17, C-19), 117.1 (C-7), 123.4 (C-15), 123.5 (C-3, C-5), 132.0 (C-16, C-20), 144.2 (C-2, C-6), 144.5 (C-14), 151.6 (C-18), 156.4 (C-4), 170.1 (C-13), 171.8 (C-25, C-28). FTMS(+) calc. for C_27_H_34_O_4_N_3_ [M+H]^+^ 464.2544 found 464.2540. UV λ_max_ (nm, CH_3_CN): 273, 490. ε (cm^−1^·M^−1^): 64,000. Φ_F_ = 0.30.

#### 3.2.7. Synthesis of 1-(5-Carboxypentyl)-4-methylquinolin-1-ium Bromide (**8**)

A mixture of 4-methylquinoline (250 mg, 1.75 mmol, 1.0 eq) and 6-bromohexanoic acid (513 mg, 2.63 mmol, 1.5 eq) in dry DMF (5 mL) was stirred at 100 °C for 24 h. The reaction was continuously monitored by TLC using CHCl_3_/CH_3_OH (8:2) as the eluent. After evaporation to dryness, the residue was purified by flash chromatography, using CHCl_3_, CHCl_3_/CH_3_OH (9:1), and CHCl_3_/CH_3_OH (8:2) as eluents, yielding 1-(5-carboxypentyl)-4-methylquinolin-1-ium bromide as an orange solid (560 mg, 95% yield). ^1^H NMR (400 MHz, CD_3_OD, δ, ppm): 1.48–1.56 (2H, m, H-12), 1.66–1.73 (2H, m, H-13), 2.07–2.14 (2H, m, H-11), 2.33 (2H, t, *J* = 7.2, H-14), 3.07 (3H, s, H-9), 5.06 (2H, t, *J* = 7.4, H-10), 7.98 (1H, d, *J*_3,2_ = 6.0, H-3), 8.06 (1H, dt, *J*_6,7_ = 8.4, H-6), 8.28 (1H, dt, *J*_7,6_ = 8.4, H-7), 8.57 (2H, dt, *J* = 9.6, H-5, H-7), 9.28 (1H, d, *J*_2,3_ = 6.0, H-2). ^13^C NMR (100 MHz, CD_3_OD, δ, ppm): 20.3 (C-9), 25.3 (C-13), 26.9 (C-12), 30.7 (C-11), 34.5 (C-14), 58.7 (C-10), 120.2 (C-3), 123.9 (C-5), 128.3 (C-8), 131.0 (C-6), 136.6 (C-7), 138.7 (C-4a), 149.2 (C-11, C-12), 160.8 (C-9), 177.3 (COOH).

#### 3.2.8. Synthesis of (*E*)-1-(5-Carboxypentyl)-4-(4-(diethylamino)styryl)quinoline-1-ium Bromide (**9**)

A mixture of compound **8** (120 mg, 0.355 mmol, 1.0 eq), 4-diethylaminobenzaldehyde (63 mg, 0.355 mmol, 1.0 eq), and piperidine (70 µL, 0.710 mmol, 2.0 eq) in dry acetonitrile (10 mL) was stirred at 85 °C for 5 h. The reaction was continuously monitored by TLC using CHCl_3_/CH_3_OH/H_2_O (65:20:2) as the eluent. The reaction mixture was evaporated to dryness and the residue purified by flash chromatography, using CHCl_3_/CH_3_OH/H_2_O (65:10:1) and CHCl_3_/CH_3_OH/H_2_O (65:20:2) as eluents, to yield (*E*)-1-(5-carboxypentyl)-4-(4-(diethylamino)styryl)quinoline-1-ium bromide as a purple solid (105 mg, 60%). ^1^H NMR (400 MHz, CD_3_OD, δ, ppm): 1.23 (6H, t, *J* = 7.1, N(CH_2_*CH*_3_)_2_), 1.46–1.50 (2H, m, H-12), 1.66–1.70 (2H, m, H-13), 2.00–2.05 (2H, m, H-11), 2.25 (2H, t, *J* = 7.2, H-14), 3.51 (4H q, *J* = 7.1, N(*CH*_2_CH_3_)_2_), 4.79 (2H, t, *J* = 7.4, H-10), 6.78 (2H, d, *J*_19,18_ = 9.0, H-19, H-21), 7.71 (2H, d, *J*_18,19_ = 9.0, H-18, H-22), 7.80 (1H, d, *J*_9,16_ = 15.5, H-9), 7.91 (1H, t, *J*_6,(5,7)_ = 8.7, H-6), 7.98 (1H, d, *J*_16,9_ = 15.5, H-16), 8.12 (1H, d, *J*_3,2_ = 6.8, H-3), 8.13 (1H, t, *J*_7,8_ = 8.7, H-7), 8.29 (1H, d, *J*_5,6_ = 8.8, H-5), 8.78 (1H, d, *J*_8,7_ = 8.8, H-8), 8.84 (1H, d, *J*_2,3_ = 6.8, H-2). ^13^C NMR (100 MHz, CD_3_OD, δ, ppm): 12.9 (C-24, C-26), 26.1 (C-13), 27.2 (C-12), 30.4 (C-11), 36.7 (C-14), 45.6 (C-23, C-25), 57.5 (C-10), 112.8 (C-19, C-21), 113.2 (C-9), 114.8 (C-3), 119.6 (C-5), 124.1 (C-17), 127.5 (C-8), 127.9 (C-4a), 129.7 (C-6), 132.9 (C-18, C-22), 136.0 (C-7), 139.6 (C-8a), 146.6 (C-2), 146.7 (C-16), 152.1 (C-20), 155.6 (C-4), 179.9 (COOH). UV λ_max_ (nm, CH_3_CN): 307, 557. ε (cm^−1^·M^−1^): 60,000. Φ_F_ = 0.02.

#### 3.2.9. Synthesis of (*E*)-1-(5-Carboxypentyl)-4-(4-(diethylamino)styryl)quinolin-1-ium Bromide (**10**)

A mixture of compound 9 (62 mg, 0.125 mmol, 1.0 eq), DCC (31 mg, 0.150 mmol, 1.2 eq), and DMAP (2 mg, 0.013 mmol, 0.1 eq) in dry DMF (1 mL) and dry acetonitrile (4 mL) was stirred at room temperature for 5 min. After this time, *N*-hydroxysuccinimide (17 mg, 0.150 mmol, 1.2 eq) was added, and the reaction was stirred at room temperature for an additional 6 h. The progress of the reaction was monitored by TLC using CHCl_3_/CH_3_OH/H_2_O (65:20:2) as the eluent. Following evaporation to dryness, the residue was purified by flash chromatography, using CHCl_3_, CHCl_3_/CH_3_OH (99:1), CHCl_3_/CH_3_OH (95:5), CHCl_3_/CH_3_OH (90:10), and CHCl_3_/CH_3_OH (80:20) as eluents, yielding (*E*)-1-(5-carboxypentyl)-4-(4-(diethylamino)styryl)quinolin-1-ium bromide as a purple solid (70 mg, 94% yield). ^1^H NMR (400 MHz, CD_3_OD, δ, ppm): 1.23 (6H, t, *J* = 7.1, N(CH_2_*CH*_3_)_2_), 1.53–1.61 (2H, m, H-12), 1.77–1.85 (2H, m, H-13), 2.01–2.09 (2H, m, H-11), 2.66 (2H, t, *J* = 7.0, H-14), 2.81 (3H, s, H-28, H-30), 3.51 (4H q, *J* = 7.1, N(*CH*_2_CH_3_)_2_), 4.81 (2H, t, *J* = 7.4, H-10), 6.78 (2H, d, *J*_19,18_ = 9.0, H-19, H-21), 7.72 (2H, d, *J*_18,19_ = 9.0, H-18, H-22), 7.80 (1H, d, *J*_9,16_ = 15.5, H-9), 7.91 (1H, t, *J*_6,(5,7)_ = 9.2, H-6), 8.03 (1H, d, *J*_16,7_ = 15.5, H-16), 8.13 (1H, dt, *J*_7,8_ = 8.7, H-7), 8.14 (1H, d, *J*_3,2_ = 6.8, H-3), 8.29 (1H, d, *J*_5,6_ = 8.9, H-5), 8.79 (1H, dd, *J*_8,7_ = 8.7, H-8), 8.84 (1H, d, *J*_2,3_ = 6.8, H-2). ^13^C NMR (100 MHz, CD_3_OD, δ, ppm): 12.9 (C-24, C-26), 25.1 (C-28, C-29), 26.4 (C-13), 26.5 (C-12), 30.0 (C-11), 31.3 (C-14), 45.6 (C-23, C-25), 57.4 (C-10), 112.8 (C-19, C-21), 113.2 (C-9), 114.9 (C-3), 119.5 (C-5), 124.1 (C-17), 127.5 (C-8), 127.9 (C-4a), 129.7 (C-6), 132.9 (C-18, C-22), 136.0 (C-7), 139.6 (C-8a), 146.6 (C-2), 146.7 (C-16), 152.1 (C-20), 155.7 (C-4), 170.1 (C-15), 171.8 (C-27, C-30). FTMS(+) calc. for C_31_H_36_O_4_N_3_ [M+H]^+^ 514.2700 found 514.2696. UV λ_max_ (nm, CH_3_CN): 307, 560. ε (cm^−1^·M^−1^): 60,000. Φ_F_ = 0.02.

### 3.3. Quantum Chemical Calculations

Computational studies offer valuable complementary insights into the nature of the electronic states of the compounds under investigation. To interpret the observed photophysical properties, density functional theory (DFT) and time-dependent density functional theory (TD-DFT) calculations were carried out using the Gaussian 16 software package [31]. The hybrid meta-GGA functional M062X [32] and the standard 6-31G(d,p) basis set were employed for optimizing the geometries of the ground state. The excited state geometries were optimized using TD-DFT with the same functional and basis set. Solvent effects were incorporated through the implicit polarized continuum model (PCM) [33,34]. Vibrational analysis showed no imaginary frequencies, confirming that the structures represent the true minima. In the TD-DFT spectral calculations, the triple-zeta 6-311+G(d,p) basis set was employed.

### 3.4. Labeling of Amino-Modified Oligonucleotides with Fluorophores

The amine-modified oligonucleotides, EUK516-mod (5′-ACCAGACTTGCCCTCC-3′), targeting the rRNA regions of eukaryotic (yeast) cells, and EUB338-mod (5′-GCTGCCTCCCGTAGGAGT-3′), targeting the rRNA regions of prokaryotic (bacterial) cells, were sourced from Eurofins Scientific (Luxembourg City, Luxembourg) and STAB Vida (Caparica, Portugal). The required amount of *N*-hydroxysuccinimide ester (NHS) was calculated based on an 8-fold molar excess as follows: mass of NHS ester [mg] = 8 × mass of modified oligonucleotide [mg] × molar mass of NHS ester [g/mol]/molar mass of modified oligonucleotide [g/mol]. The total reaction volume was adjusted to achieve a final concentration of 200 μM. The NHS ester was dissolved in DMSO, constituting 1/10 of the total reaction volume, while the modified oligonucleotide was dissolved in a 0.1 M sodium bicarbonate solution, making up the remaining 9/10 of the total reaction volume. The NHS ester solution was introduced into the modified oligonucleotide solution and thoroughly stirred for 1 min using a vortex. The reaction mixture was stirred on an orbital shaker for 12 h at 20 °C, protected from light. Next, 1/10 volume of 3 M NaCl and 2.5 volumes of cold (−20 °C) absolute ethanol were added to the reaction mixture. The mixture was vortexed thoroughly and then incubated at −20 °C for 30 min. Following incubation, the reaction mixture was centrifuged at 12,000× *g* for 30 min, and the supernatant was carefully removed. The resulting fluorescent bioconjugate pellet was washed four times with 70% ethanol (4 × 1 mL) and then air-dried [35].

### 3.5. RNA-FISH Methodology

Universal probes were used as hybridization controls as follows: EUB338-Cy3 (5′Cy3-GCTGCCTCCCGTAGGAGT-3′), which targets the rRNA regions of prokaryotic (bacterial) cells, and EUK516-Cy3 (5′Cy3-ACCAGACTTGCCCTCC-3′), which targets the rRNA regions of eukaryotic (yeast) cells. These probes feature a cyanine (Cy3^®^) fluorescent label attached to the 5′ end of the oligonucleotide. The solutions required for the fluorescence in situ hybridization (FISH) procedure, specifically phosphate-buffered saline (PBS 10X) containing NaCl (130.0 mM), NaH_2_PO_4_ (8.0 mM), KCl (2.7 mM), KH_2_PO_4_ (1.5 mM) at pH 7.2, and the hybridization buffer (HB) consisting of NaCl (0.9 M), Tris-HCl (20 mM), and SDS (0.1%), were prepared in advance. The RNA-FISH methodology involves several preparatory steps, including: (i) fixation of sample cells, (ii) permeabilization of cells, (iii) hybridization with specific probes to target the desired sequences, (iv) washing to eliminate unbound probes, and (v) detection and quantification of signals [36]. The two following microorganisms were chosen as biological models: (i) the yeast *Saccharomyces cerevisiae* and (ii) the bacteria *Bacillus* sp., both sourced from the culture collection of the Laboratory of Biodegradation and Biotechnology at the HERCULES Laboratory, University of Évora. Bacterial and yeast cells were initially cultivated for 2 days at 30 °C on nutrient agar (composed of 5.0 g/L peptone, 3.0 g/L beef extract powder, and 15.0 g/L agar, pH 7.4) and YPD-agar (composed of 20.0 g/L glucose, 20.0 g/L peptone, 10.0 g/L yeast extract, and 20.0 g/L agar, pH 6.0) slants, respectively. They were then stored at 4.0 °C. To prepare the inocula, cells were harvested from the fresh surfaces of the slants using 2.0 mL of physiological saline solution. Subsequently, 250 mL Erlenmeyer flasks containing 100 mL of nutrient broth (NB) and YPD were inoculated and incubated at 30 °C with continuous shaking at 120 rpm. Cells were collected during the exponential growth phase, which occurred 6 h after inoculation, and then washed with PBS. **Preparation of the probe solution** (**oligonucleotide-labeling**)**.** The products obtained from the labeling reaction between the oligonucleotides and the probes were reconstituted in RNase-free water, resulting in solutions with a final concentration of 120 ng/μL. **Cell fixation and permeabilization.** The microbial cultures of *Saccharomyces cerevisiae* and *Bacillus* sp. were initially prepared in liquid medium and then centrifuged at 1600× *g* for 5 min at 4 °C and 130,00× *g* for 15 min at 4 °C, respectively, discarding the supernatant. The cells were washed by adding 3.0 mL of PBS 10X and centrifuged under the same conditions, again discarding the supernatant. Next, the cell pellets were fixed and permeabilized by adding 2.0 mL of absolute ethanol and incubating at room temperature for 1 h. After incubation, 6.0 mL of PBS 10X was added. The cells were then resuspended in a 50:50 mixture of ethanol and PBS 10X and stored in the freezer at −20.0 °C. **Cell count.** The fixed yeast and bacterial cells were thawed and centrifuged at 1600× *g* for 5 min at 4 °C and 13,000× *g* for 15 min at 4 °C, respectively, after which the supernatant was discarded. The cells were then resuspended in 1.0 mL of PBS 10X. Next, in a microtube, 10.0 µL of the cell suspension was mixed with 990.0 µL of methylene blue, vortexed, and incubated at room temperature for 15 min. The stained cells were counted using a Neubauer chamber to calculate the volume of the cell suspension. **Hybridization.** For hybridization, microtubes were prepared with equal aliquots containing 10^6^ yeast or 10^8^ bacterial cells, which had been previously fixed. After centrifugation at 1600× *g* for 5 min at 4 °C for yeast cells and at 13,000× *g* for 15 min at 4 °C for bacterial cells, 80 μL of hybridization buffer (HB) composed of 0.9 M NaCl, 20 mM Tris-HCl, 0.1% SDS (pH 7.2) was added. The corresponding volume of the RNA-FISH probe stock solution (120 ng/μL) was then added to each FISH assay. The samples were mixed and incubated in a water bath at 46 °C for 2 h. The FISH assays included the following: (i) a blank without added probe, (ii) controls for natural autofluorescence and FISH-induced autofluorescence, and (iii) controls with added EUK516-(**4**), EUK516-(**7**), and EUK516-(**10**) (universal probes for eukaryotes, serving as the positive control for yeast), as well as EUB338-(**4**), EUB338-(**7**), and EUB338-(**10**) (universal probes for bacteria, serving as the positive control for bacteria and the negative control for yeast). **Washing.** After hybridization, the cells were washed with 100 μL of hybridization buffer (HB) and then incubated in a water bath at 46 °C for 30 min. Subsequently, after centrifugation at 1600× *g* for 5 min at 4 °C (yeast cells) and 13,000× *g* for 15 min at 4 °C (bacterial cells), cells were resuspended in 400 μL of PBS 10x. The samples were then examined using epifluorescence microscopy (EM) and flow cytometry (FC), following the protocol outlined by Branco et al. [37].

### 3.6. Epifluorescence Microscopy (EM) and Flow Cytometry (FC) Analysis

For microscopic analysis, small portions of the samples were placed onto the microscope slides. These samples were then examined using an epifluorescence microscope (Motic BA410E) equipped with a 100 W quartz halogen Köhler illumination system that features intensity control, an epi-attachment EF-UPR-III, and a power supply unit MXH-100, all sourced from Motic (Madrid, Spain). To facilitate this analysis, Motic filters specifically designed for TRITC were employed, with an excitation wavelength of 540/40×, a 565DCLP dichroic mirror, and a 605/55m barrier filter. Microphotographs were captured using a Moticam PRO 282B camera connected to the microscope, and the images were subsequently analyzed using Motic Images Plus 2.0^LM^ software (Motic, Madrid, Spain). For flow cytometry (FC) analysis, the Muse^®^ Cell Analyzer (Merck KGaA, Darmstadt, Germany) was used along with MuseSoft 1.4.0.0 software. For each RNA-FISH assay, the percentage of cells that became fluorescent after FISH treatment was assessed, as well as their fluorescence intensities (FI), using the red (680/30) photodiode detector. A total of 1000 events were acquired for this analysis. FI values were recorded based on a gate defined in a FI versus Forward Scatter (FSC) density plot, considering the results from blanks, controls, and test assays. The fluorescence intensity conferred by the probe (FIpro), which is the sum of the fluorescence intensities of cells detected after RNA-FISH treatment with either of the probes (EUB338 or EUK516), was directly calculated from the flow cytometer results. Each sample was analyzed in triplicate.

## 4. Conclusions

In summary, our aim to produce affordable and effective fluorescent labels for biomolecules led us to synthesize new small molecules using an efficient and cost-effective strategy starting with 4-diethylaminobenzaldehyde. Extending the π-conjugated system in these molecules was essential to facilitate the effective intramolecular charge transfer process of the emissive excited state, improving their photophysical properties, including high molar extinction coefficients and large Stokes shifts. The new fluorescent labels demonstrate high efficacy as RNA-FISH probes, enabling the specific detection of microbial cells. Some of the synthesized probes exhibited a similar proportion of fluorescent cells comparable to Cy3^®^, one of the most widely used, and expensive, commercial fluorescent labels. The slightly lower efficiency observed in the synthesized probes derived from the fluorescent markers containing derivative **10** may be due to the low value of its fluorescence quantum yield. Considering that FISH is a crucial cytogenetic technique widely used in genomic and cell biological research, as well as in diagnostic applications in preventive and reproductive medicine and oncology, these new affordable and effective fluorescent labels hold significant potential for routine use in these fields. Beyond the FISH technique, these findings provide groundwork for exploring a wide range of potential applications for these promising new amino-reactive fluorescent labels. The use of the DFT and TD-DFT calculations provided valuable insights into the observed photophysical properties, particularly the intramolecular charge transfer and large Stokes shifts. These shifts were attributed to the significant structural relaxation of the molecules in the excited states, offering a rational explanation for the phenomenon.

## Figures and Tables

**Table 1:** Spectroscopic properties of 4-styryl-*N*,*N*-diethylaniline derivatives.

Compounds	λ_abs_^a^ (nm)	λ_em_^b^ (nm)	Stokes Shift (nm, cm^−1^)	ε ^c^ (cm^−1^·M^−1^)	Φ_F_^d^
**1**	342	--	--	19,400	--
**4**	556	597	41, 1235	90,000	0.57
**7**	490	604	114, 3852	64,000	0.30
**10**	560	665	105, 2820	60,000	0.02

^a^ Absorption maxima in acetonitrile; ^b^ Emission maxima in acetonitrile; ^c^ Molar extinction coefficient at longest wavelength transition; ^d^ Fluorescence quantum yield in ethanol, determined using 7-diethylamino-4-methylcoumarin (Φ_F_ = 0.73 in ethanol) as standard.

## Data Availability

Computational files are available from the authors.

## Supplementary Materials

The following supporting information can be downloaded at: https://www.mdpi.com/article/10.3390/molecules29225237/s1, Figure S1. Optimized molecular geometry for the compounds in acetonitrile at the m06-2x/6-31G(d,p) level (left) where oxygen, carbon, nitrogen, and hydrogen atoms are marked in red, gray, blue, and white, respectively. Contour plots of the electron density difference (Δρ) of the lowest energy excitation for the different compounds where magenta indicates an increase in electron density, while green represents a depletion of electron density; Figure S2. Flow cytometry (FC) results (fluorescence intensity (FI)/forward scattering (FSC)) referring to the hybridization assays of *Saccharomyces cerevisiae* cells with the oligonucleotide probes: (A) EUK516-Cy3; (B) EUB338-Cy3; (C) EUK516-(**4**); (D) EUB338-(**4**); (E) EUK516-(**7**); (F) EUB338-(**7**); (G) EUK516-(**10**); (H) EUB338-(**10**); Figure S3 Microphotographs obtained through epifluorescence microscopy in objective amplification of 50 X with the TRITC filter referring to the hybridization assays of *Saccharomyces cerevisiae* cells with the oligonucleotide probes: (A) EUK516-Cy3; (B) EUB338-Cy3; (C) EUK516-(**4**); (D) EUB338-(**4**); (E) EUK516-(**7**); (F) EUB338-(**7**); (G) EUK516-(**10**); (H) EUB338-(**10**); Figure S4. Flow cytometry (FC) results (fluorescence intensity (FI)/forward scattering (FSC)) referring to the hybridization assays of *Bacillus* sp. cells with the oligonucleotide probes: (A) EUB338-Cy3; (B) EUK516-Cy3; (C) EUB338-(**4**); (D) EUK516-(**4**); (E) EUB338-(**7**); (F) EUK516-(**7**); (G) EUB338-(**10**); (H) EUK516-(**10**); Figure S5. Microphotographs obtained through epifluorescence microscopy in objective amplification of 100 X with the TRITC filter referring to the hybridization assays of *Bacillus* sp. cells with the oligonucleotide probes: (A) EUB338-Cy3; (B) EUK516-Cy3; (C) EUB338-(**4**); (D) EUK516-(**4**); (E) EUB338-(**7**); (F) EUK516-(**7**); (G) EUB338-(**10**); (H) EUK516-(**10**). Table S1. Calculated absorption data for the compounds, the main orbitals involved in the transitions and the CT characterization indexes; Table S2. Rings planes angle (°) and BLA (Å) of the compounds in the ground S_0_ and excited S_1_ states; Table S3. Calculated emission data for the studied compounds, the main orbitals involved in the S_1_→S_0_ transitions and the theoretical fluorescent lifetime of the excited states.
