# Supplementary material for: Affordable Small Molecules as Promising Fluorescent Labels for Biomolecules
Source: Molecules. 2024 Nov 5;29(22):5237. doi: 10.3390/molecules29225237 (PMC11596234; doi:10.3390/molecules29225237)
Supplement: Supplementary file 1 [file molecules-29-05237-s001.zip › molecules-3242720-supplementary.pdf]

Supplementary Information (SI)

Contents:

1 NMR spectra 1
2 Mass spectra 7
3 UV/Vis spectra 9
4 Quantum chemical calculation 10
5 RNA-FISH performance of the synthesized oligonucleotide probes 13

1 NMR spectra

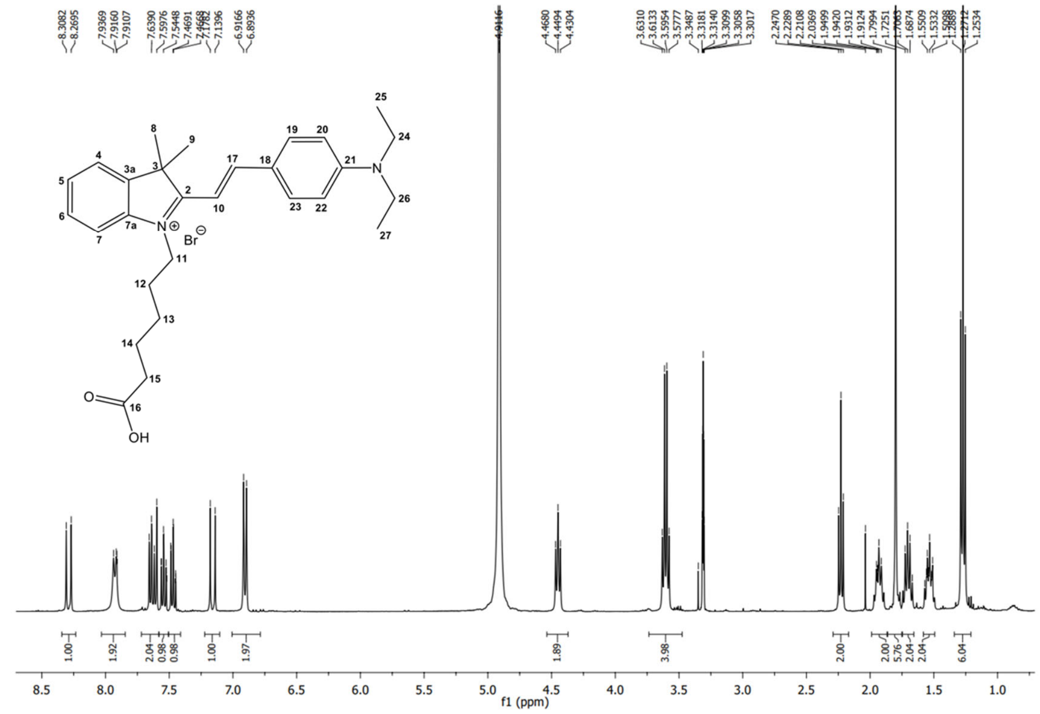

*^{1}H-NMR spectrum of **3** (CD$_3$OD, 400 MHz)*

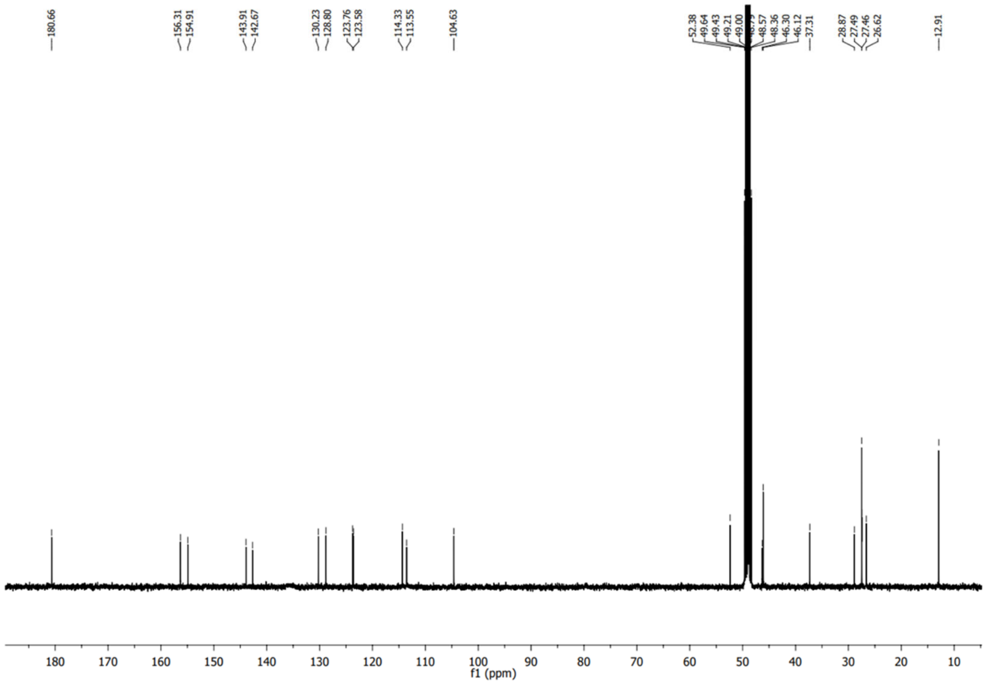

*^{13}C-NMR spectrum of **3** (CD$_3$OD, 100 MHz)*

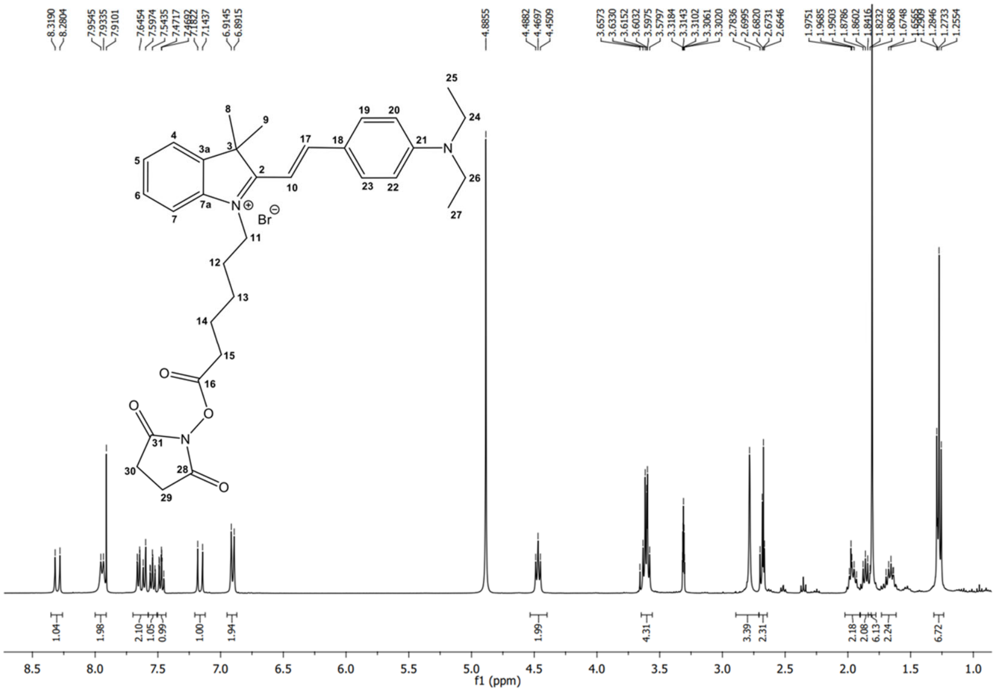

*^{1}H-NMR spectrum of **4** (CD_3OD, 400 MHz)*

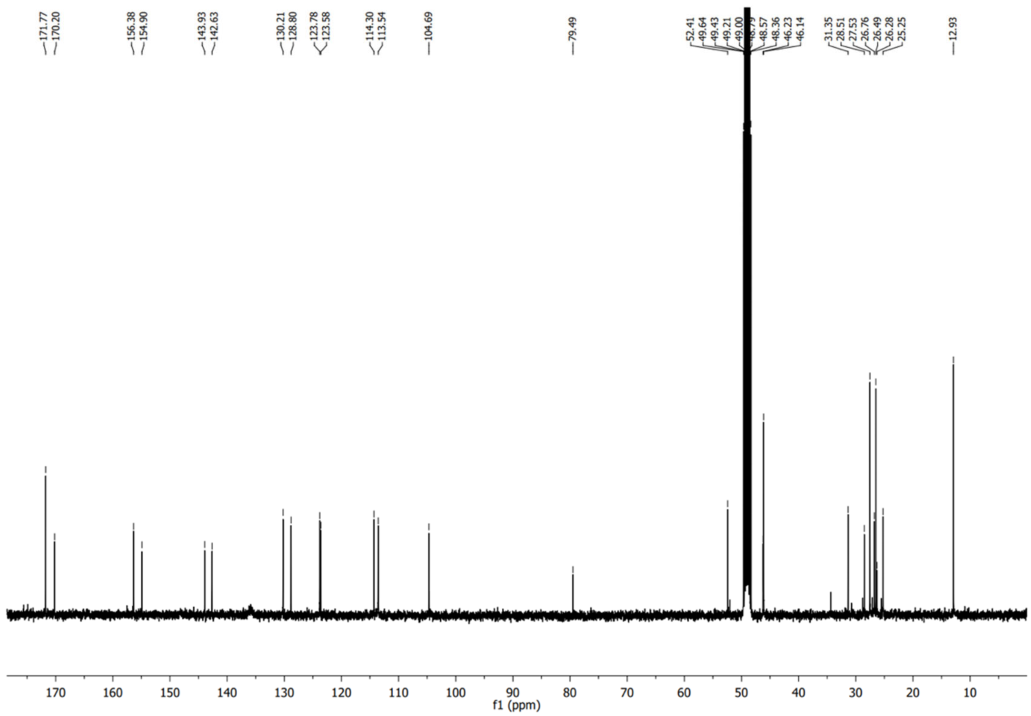

*^{13}C-NMR spectrum of **4** (CD_3OD, 100 MHz)*

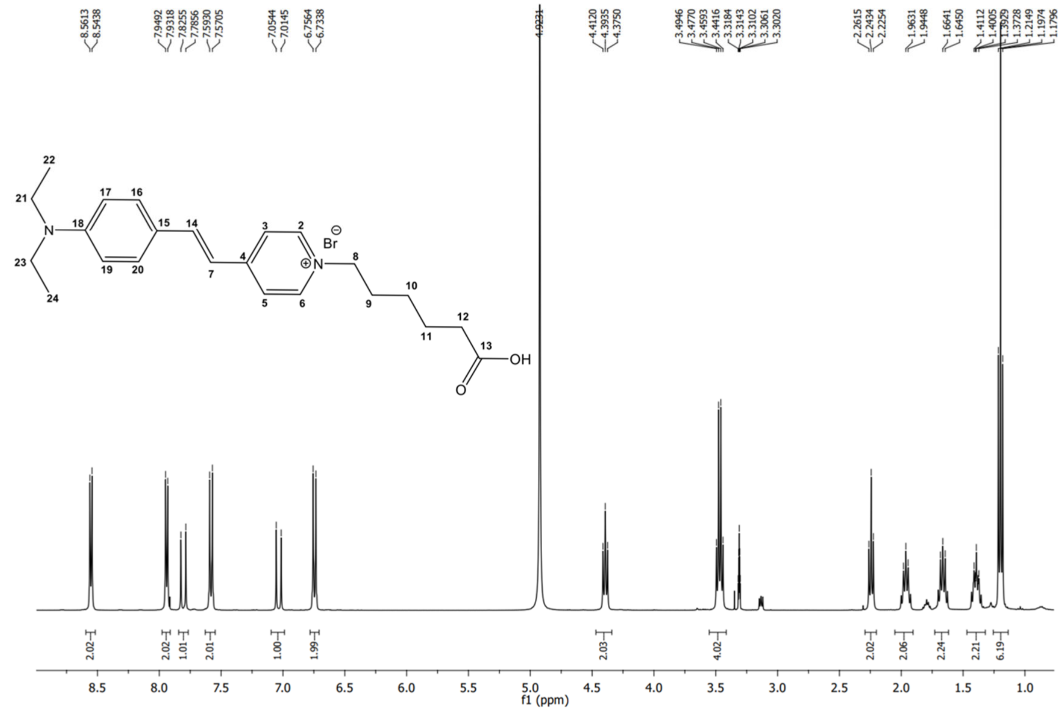

*^{1}H-NMR spectrum of **6** (CD_3OD, 400 MHz)*

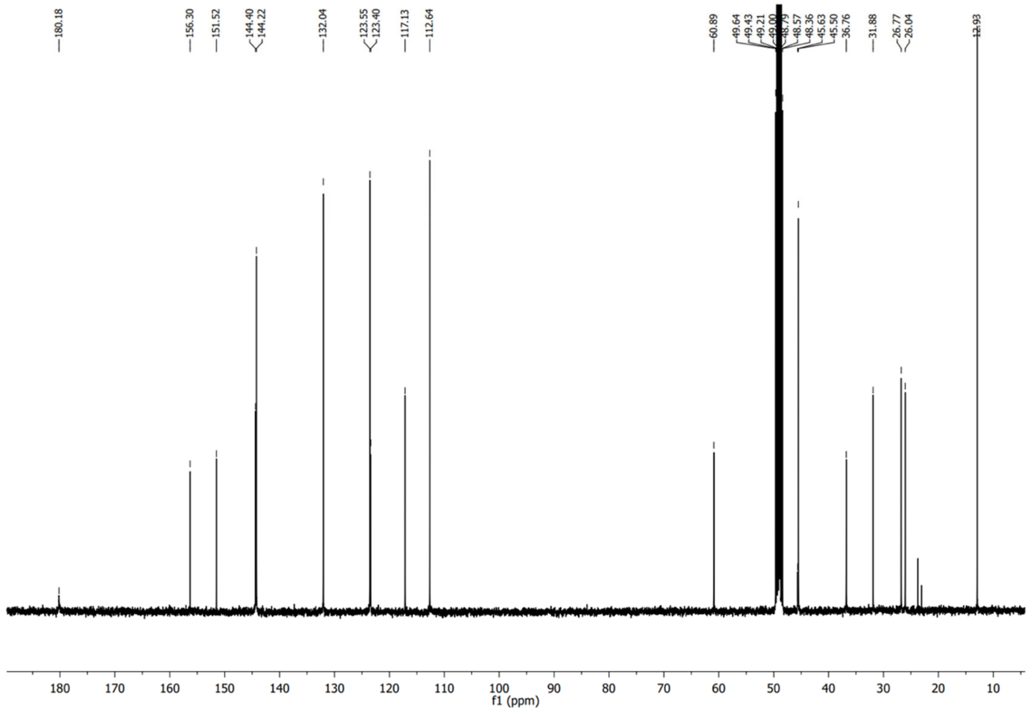

*^{13}C-NMR spectrum of **6** (CD_3OD, 100 MHz)*

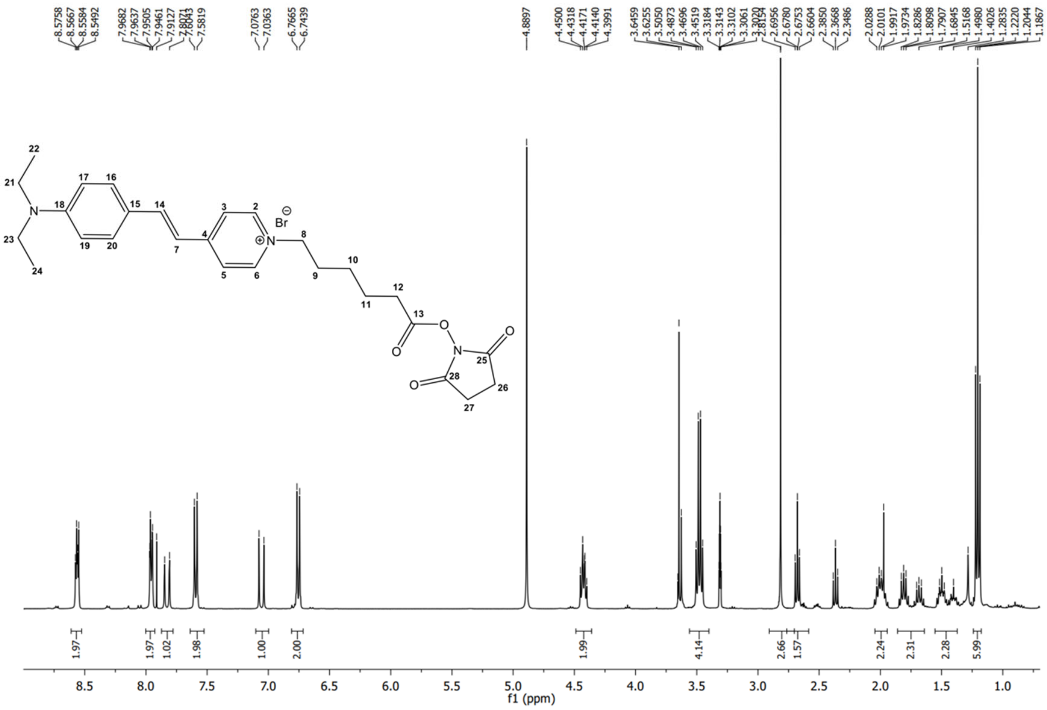

^{1}H-NMR spectrum of ***7*** *(CD₃OD, 400 MHz)*

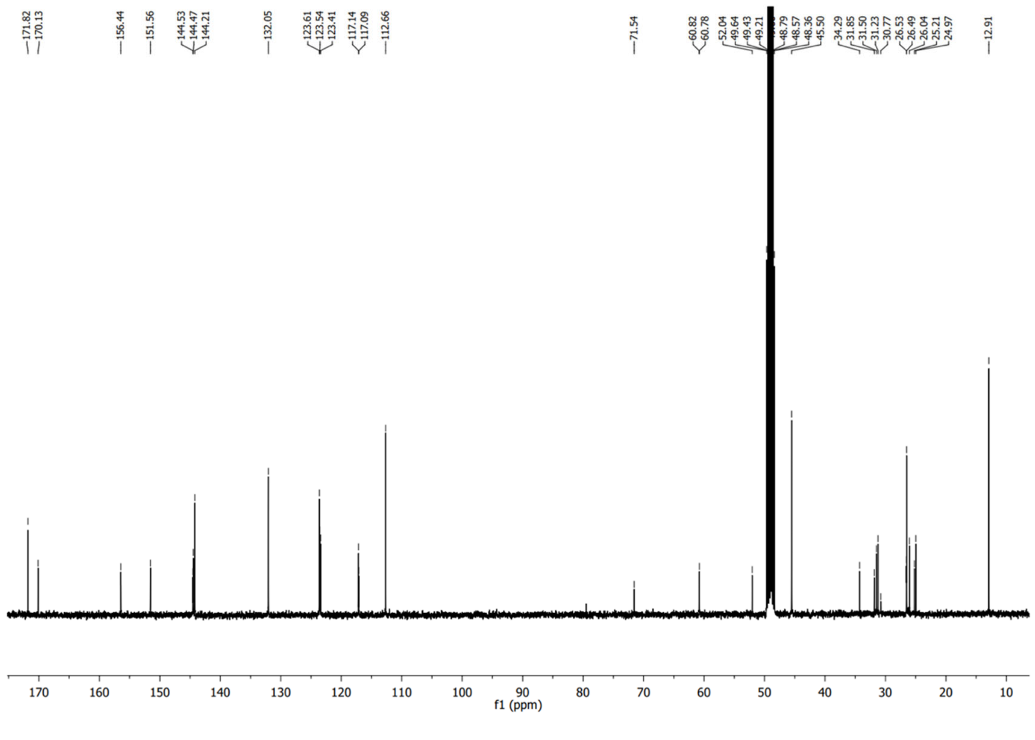

^{13}C-NMR spectrum of ***7*** *(CD₃OD, 100 MHz)*

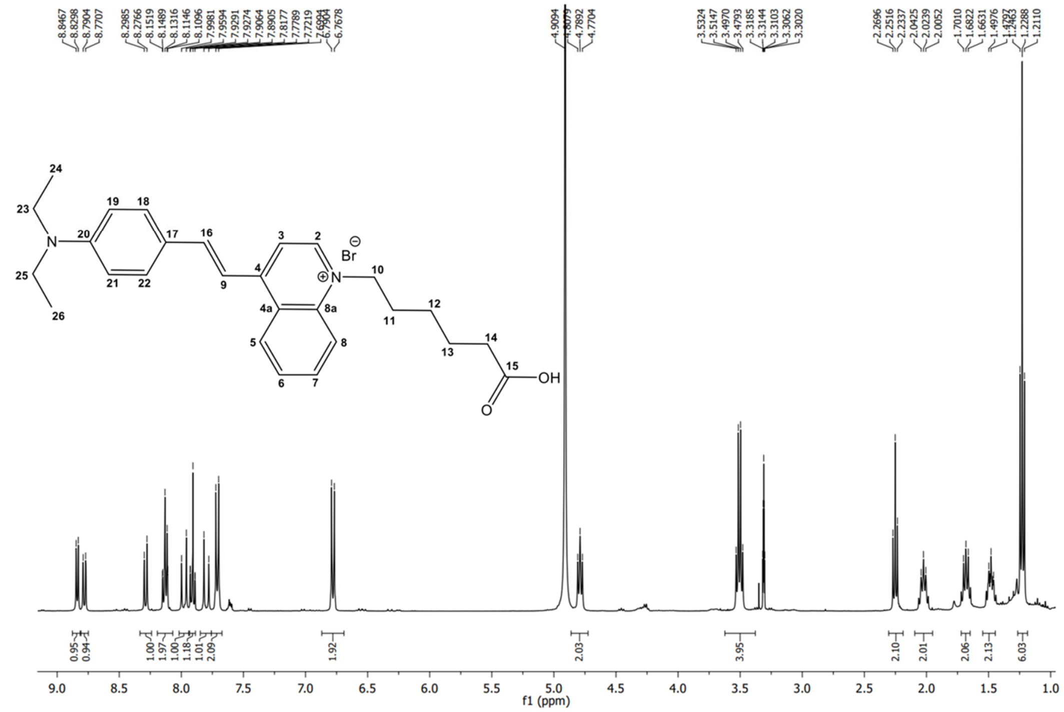

*^{1}H-NMR spectrum of **9** (CD_3OD, 400 MHz)*

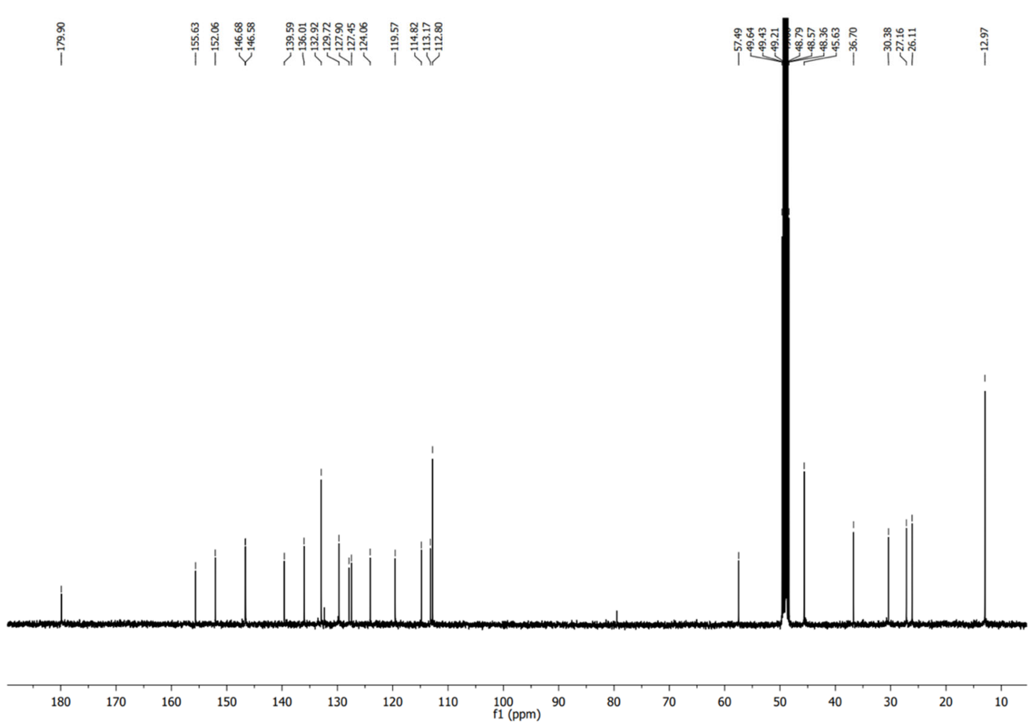

*^{13}C-NMR spectrum of **9** (CD_3OD, 100 MHz)*

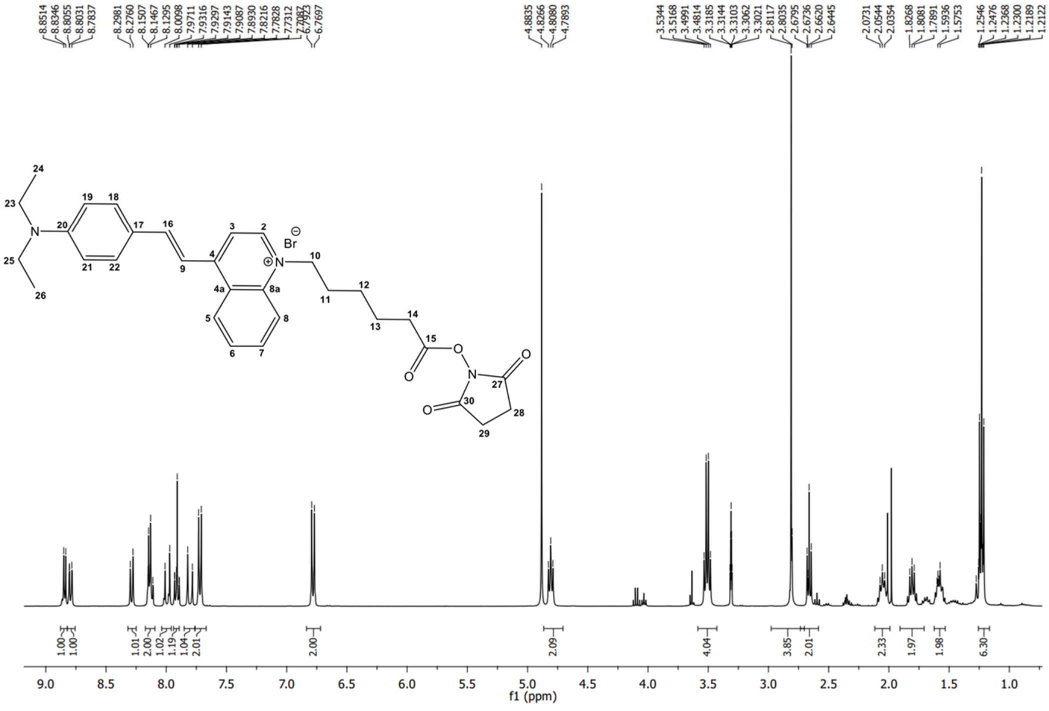

*^{1}H-NMR spectrum of **10** (CD3OD, 400 MHz)*

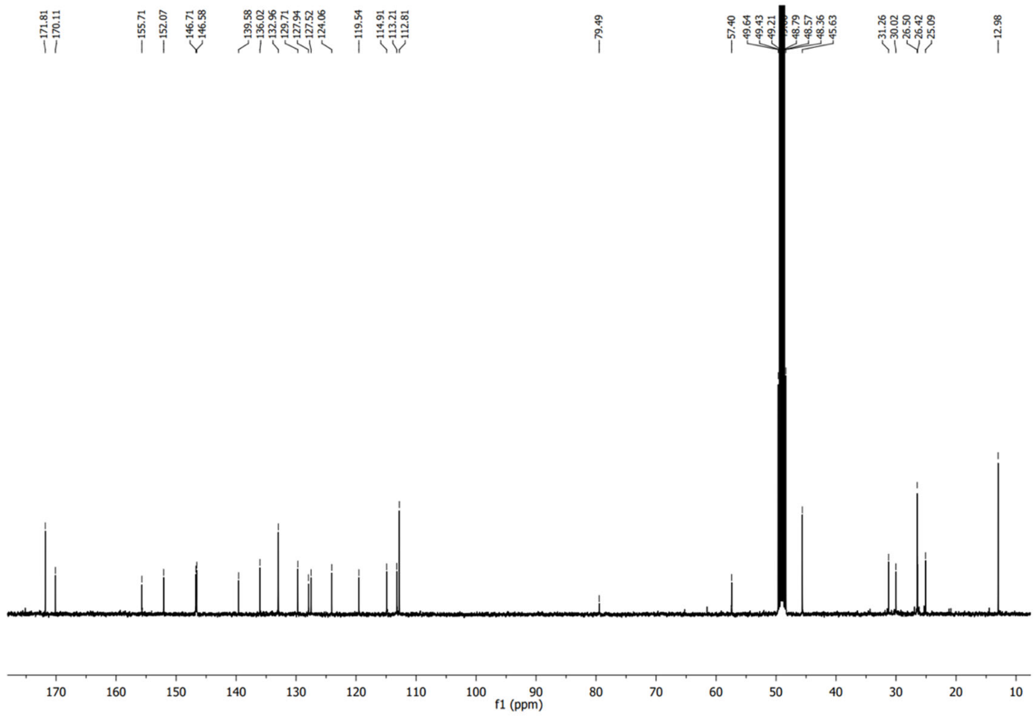

*^{13}C-NMR spectrum of **10** (CD3OD, 100 MHz)*

2 Mass spectra

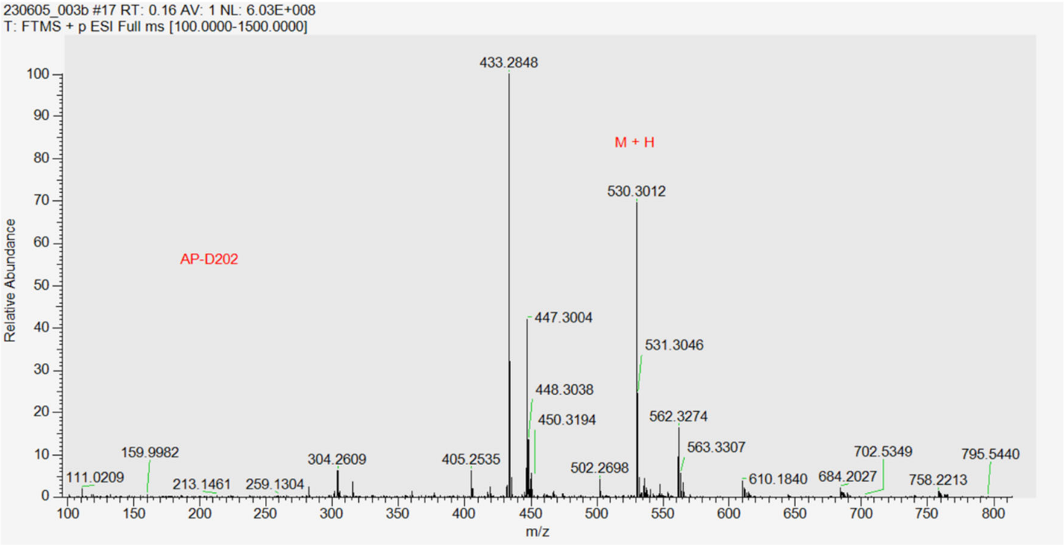

Mass spectrum of ***4***

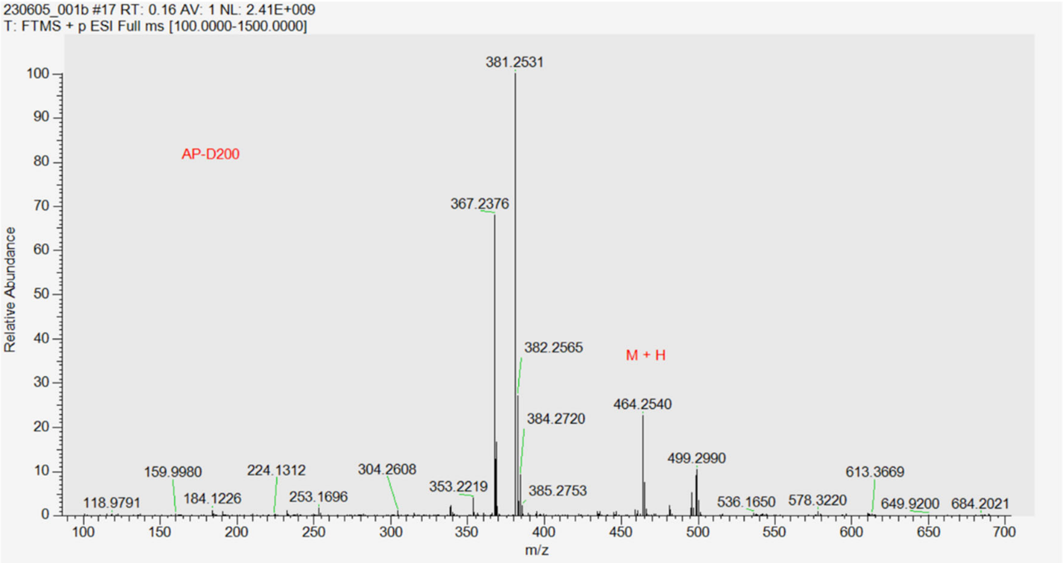

Mass spectrum of ***7***

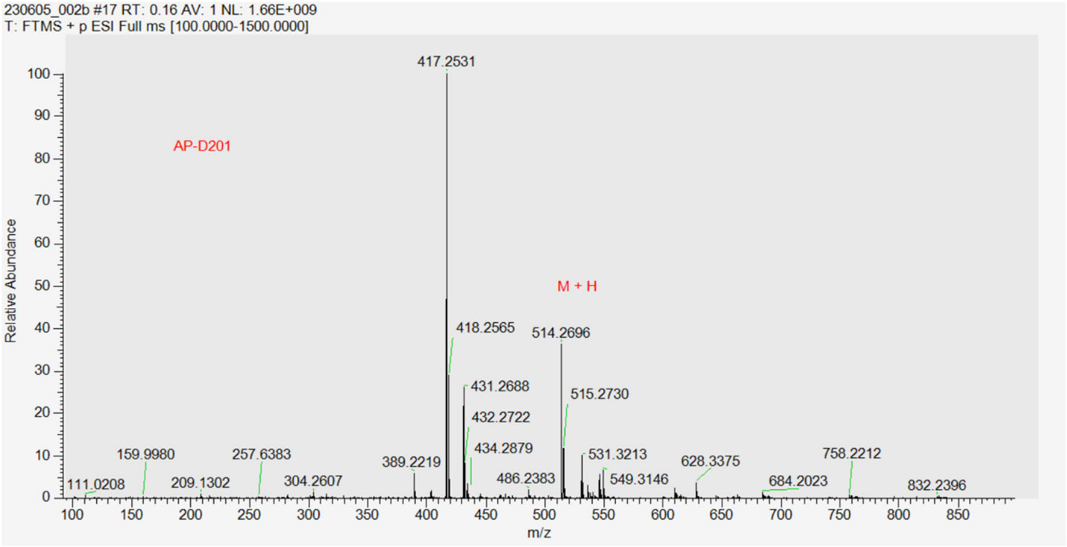

*Mass spectrum of **10***

3 UV/Vis spectra

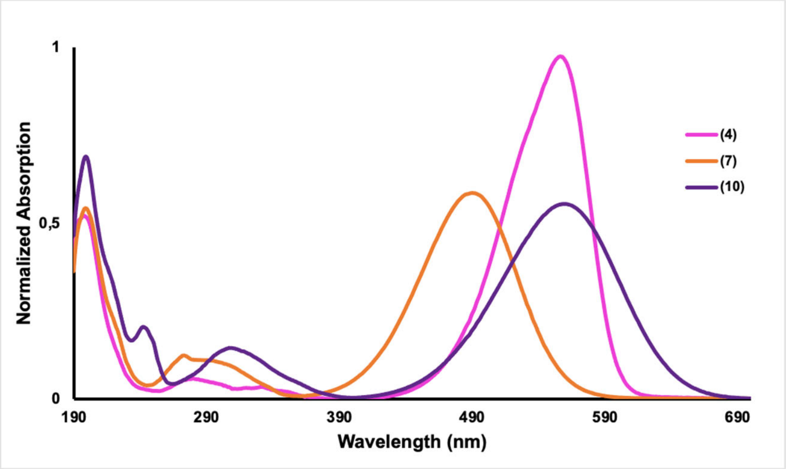

*UV/Vis spectra of **4, 7** and **10***

4 Quantum chemical calculation

4

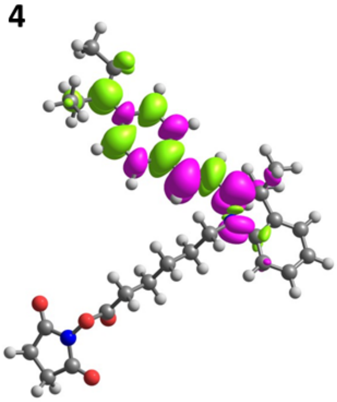

7

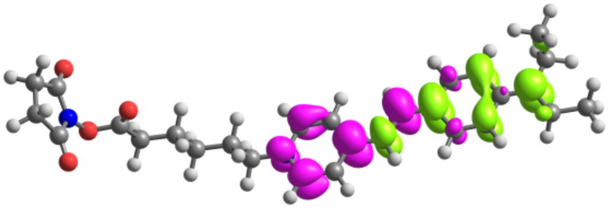

10

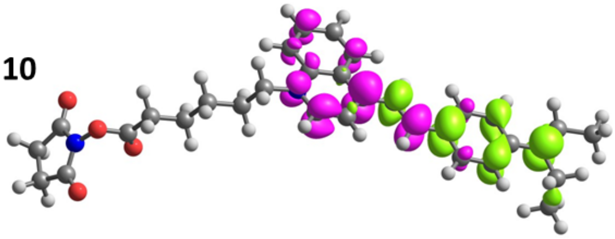

Figure S1. Optimized molecular geometry for the compounds in acetonitrile at the m06-2x/6-31G(d,p) level (left) where oxygen, carbon, nitrogen and hydrogens atoms are marked in red, gray, blue and white, respectively. Contour plots of the electron density difference (Δϱ) of the lowest energy excitation for the different compounds where magenta indicates an increase of electron density while green represents a depletion of electron density.

Table S1. Calculated absorption data for the compounds, the main orbitals involved in the transitions and the CT characterization indexes.

Compound 4

State	λ (nm)	f	Major MO → MO contributions	$\Delta\mathbf{r}$/Å	Λ
S_1	502	1.7786	HOMO→LUMO (96%)	2.18	0.65
S_2	299	0.0088	H-1→LUMO (92%)		
S_3	281	0.0015	H-3→LUMO (57%), HOMO→L+3 (11%), HOMO→L+6 (13%)		

Compound 7

State	λ (nm)	f	Major MO → MO contributions	$\Delta\mathbf{r}$/Å	Λ
S_1	476	1.6509	HOMO→LUMO (94%)	4.07	0.58
S_2	286	0.0472	HOMO→L+1 (47%), HOMO→L+3 (18%), HOMO→L+7 (16%)		
S_3	279	0.0111	HOMO→L+1 (39%), HOMO→L+3		

Compound 10

State	λ (nm)	f	Major MO → MO contributions	$\Delta\mathbf{r}$/Å	Λ
S_1	534	1.6323	HOMO→LUMO (93%)	4.04	0.58
S_2	315	0.0041	H-1→LUMO (79%)		
S_3	294	0.0834	HOMO→L+1 (57%), HOMO→L+6 (14%)		

Table S2. Rings planes angle (°) and BLA (Å) of the compounds in the ground S_0 and excited S_1 states.

	BLA		Angle	
**Compound**	S_0	S_1	S_0	S_1
**4**	0.04	0.00	19.4	16.6
**7**	0.07	0.01	0.3	1.1
**10**	0.06	0.02	8.1	1.1

Table S3. Calculated emission data for the studied compounds, the main orbitals involved in the $S_1 \rightarrow S_0$ transitions and the theoretical fluorescent lifetime of the excited states.

**Compound**	λ (nm)	f	Major MO → MO contributions	τ / ns
**4**	544	1.7889	HOMO→LUMO (99%)	2.48
**7**	533	1.7778	HOMO→LUMO (99%)	2.39
**10**	587	1.7436	HOMO→LUMO (99%)	2.96

RNA-FISH performance of the synthesized oligonucleotide probes

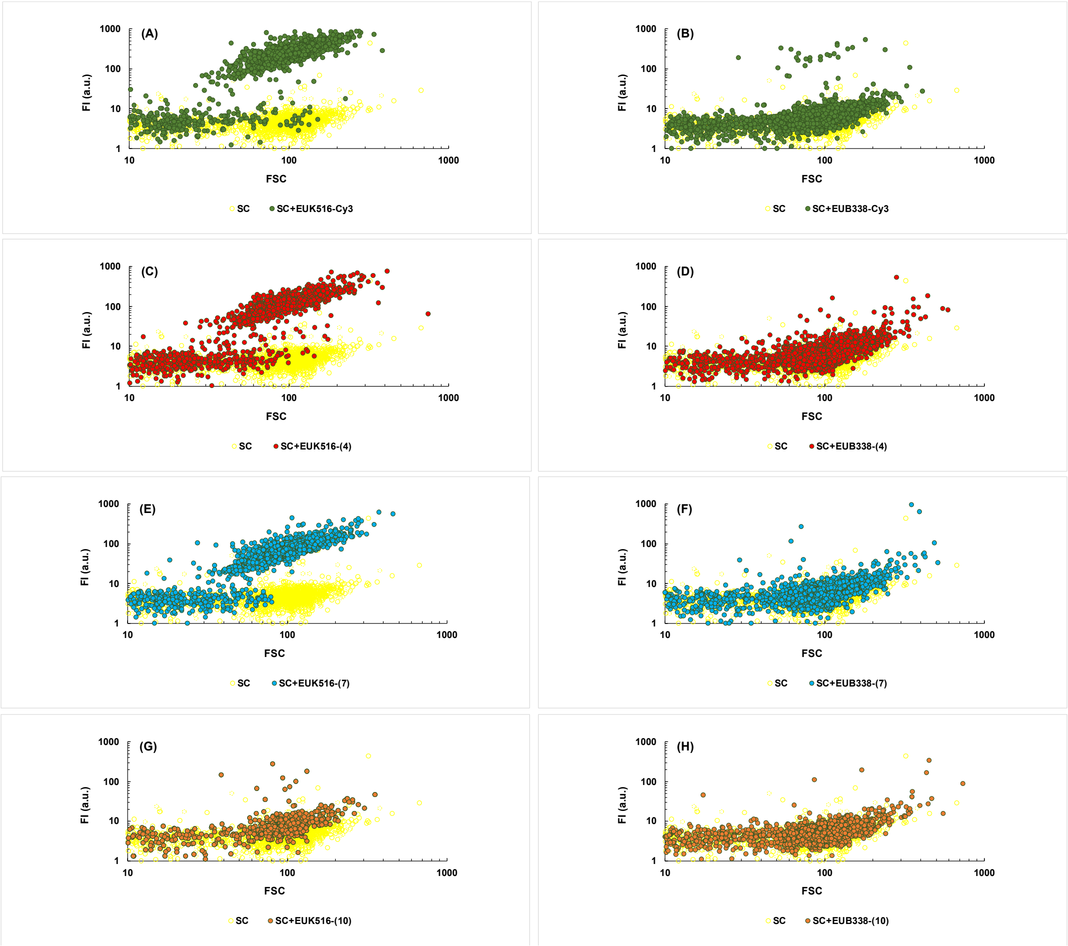

Figure S2 Flow cytometry (FC) results (fluorescence intensity (FI)/forward scattering (FSC)) referring to the hybridization assays of *Saccharomyces cerevisiae* cells with the oligonucleotide probes: **(A)** EUK516-Cy3; **(B)** EUB338-Cy3; **(C)** EUK516-(**4**); **(D)** EUB338-(**4**); **(E)** EUK516-(**7**); **(F)** EUB338-(**7**); **(G)** EUK516-(**10**); **(H)** EUB338-(**10**).

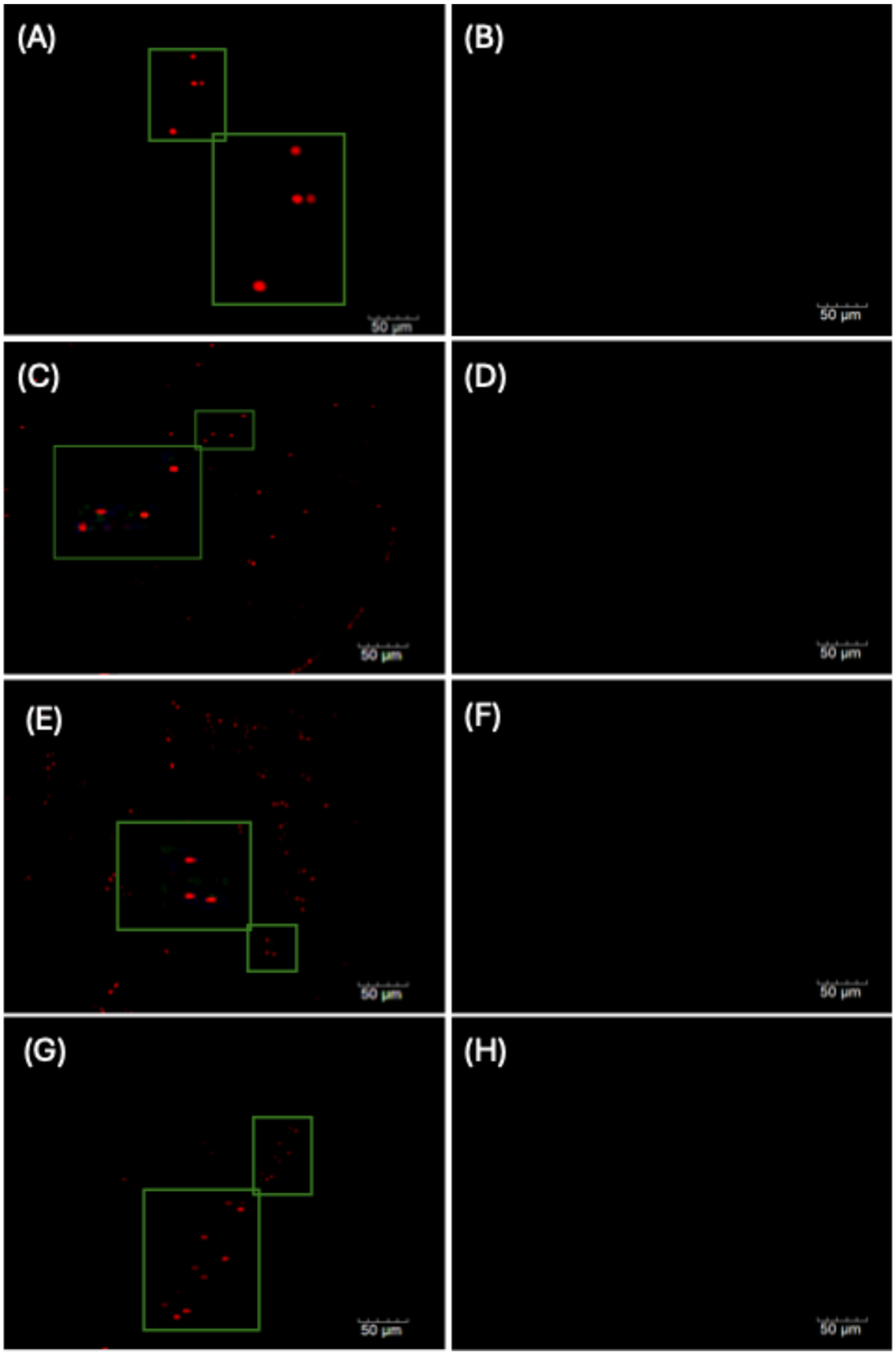

Figure S3 Microphotographs obtained through epifluorescence microscopy in objective amplification of 50 X with the TRITC filter referring to the hybridization assays of *Saccharomyces cerevisiae* cells with the oligonucleotide probes: **(A)** EUK516-Cy3; **(B)** EUB338-Cy3; **(C)** EUK516-(**4**); **(D)** EUB338-(**4**); **(E)** EUK516-(**7**); **(F)** EUB338-(**7**); **(G)** EUK516-(**10**); **(H)** EUB338-(**10**).

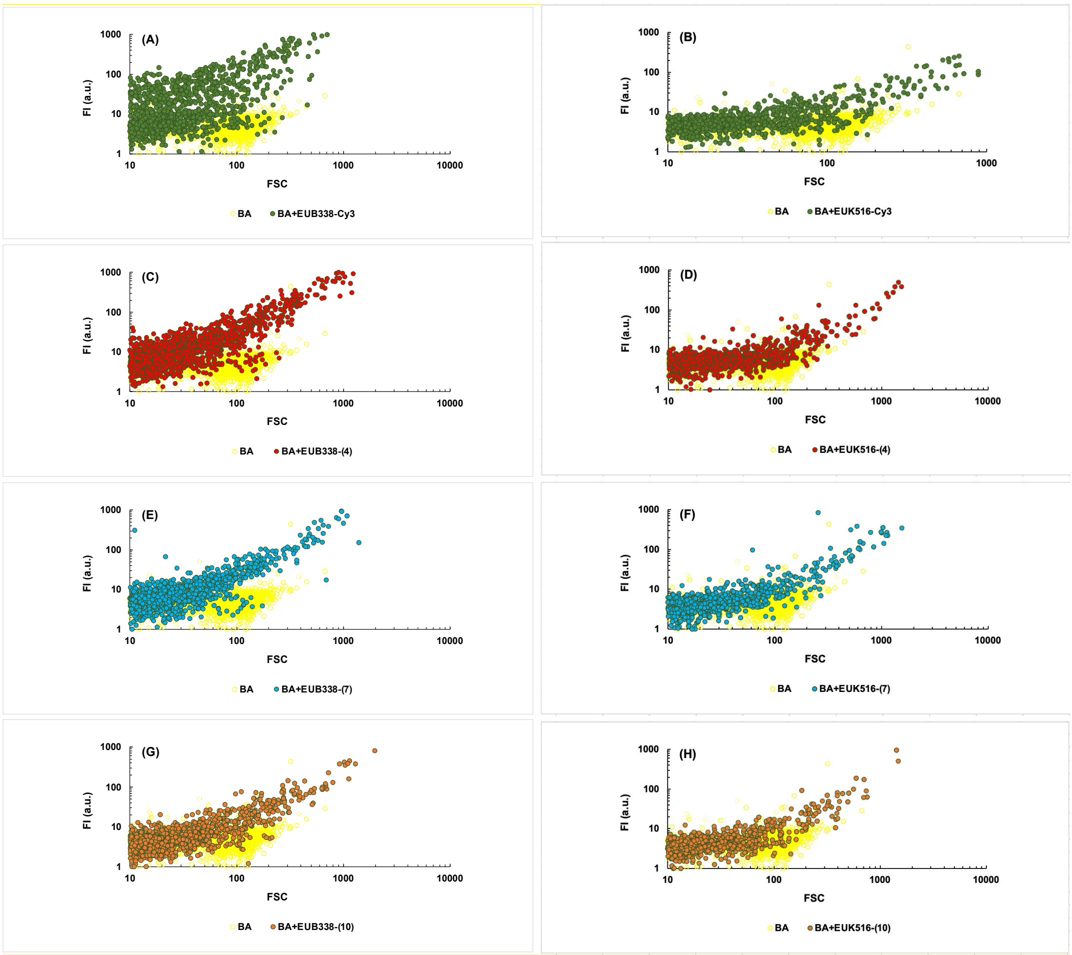

Figure S4 Flow cytometry (FC) results (fluorescence intensity (FI)/forward scattering (FSC)) referring to the hybridization assays of *Bacillus* sp. cells with the oligonucleotide probes: **(A)** EUB338-Cy3; **(B)** EUK516-Cy3; **(C)** EUB338-(**4**); **(D)** EUK516-(**4**); **(E)** EUB338-(**7**); **(F)** EUK516-(**7**); **(G)** EUB338-(**10**); **(H)** EUK516-(**10**).

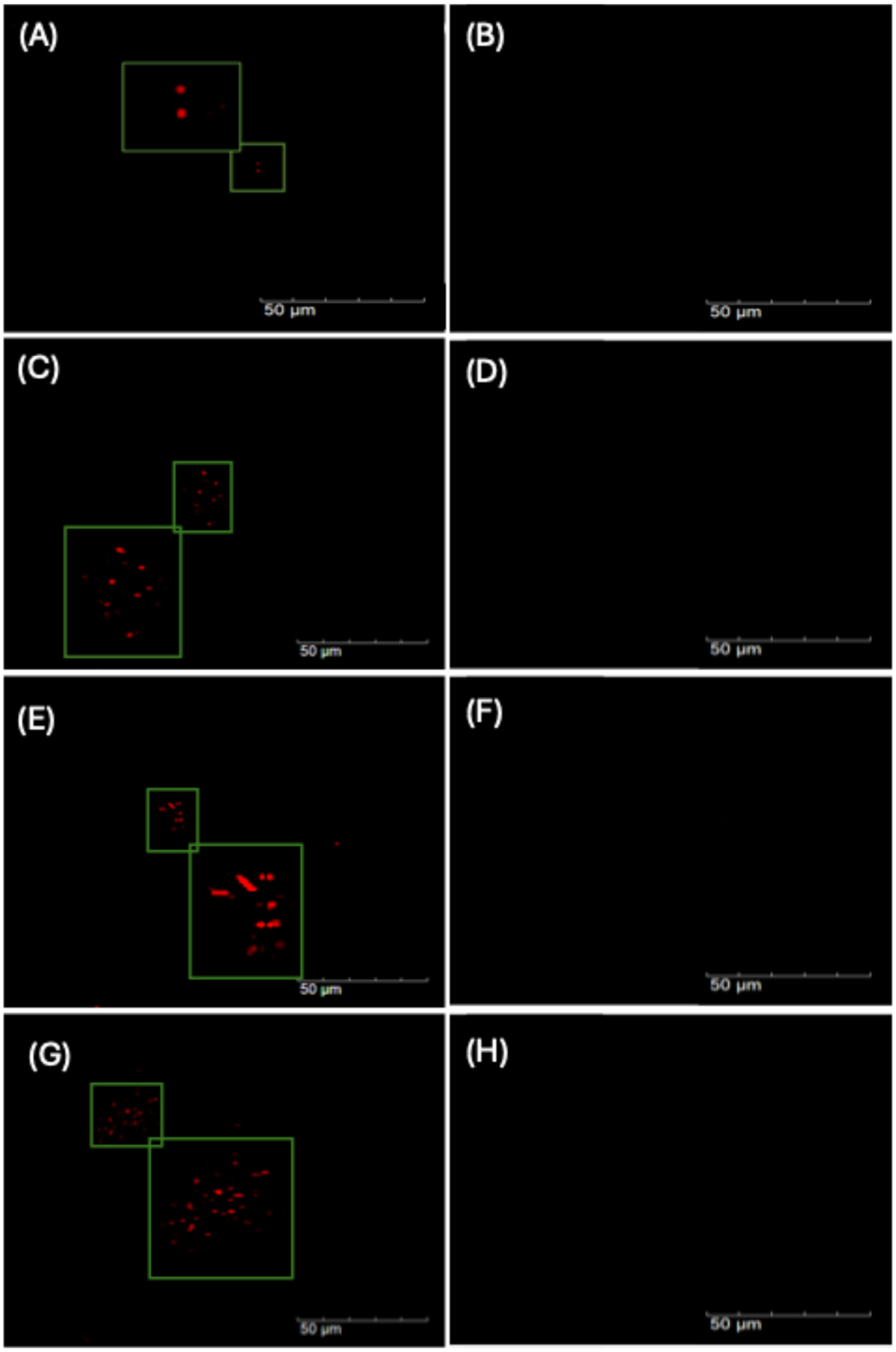

Figure S5 Microphotographs obtained through epifluorescence microscopy in objective amplification of 100 X with the TRITC filter referring to the hybridization assays of *Bacillus* sp. cells with the oligonucleotide probes: **(A)** EUB338-Cy3; **(B)** EUK516-Cy3; **(C)** EUB338-(**4**); **(D)** EUK516-(**4**); **(E)** EUB338-(**7**); **(F)** EUK516-(**7**); **(G)** EUB338-(**10**); **(H)** EUK516-(**10**).
